# The genetic basis of replicated bullseye pattern reduction across the *Hibiscus trionum* complex

**DOI:** 10.1111/nph.70168

**Published:** 2025-05-20

**Authors:** May T. S. Yeo, Alice L. M. Fairnie, Valentina Travaglia, Joseph F. Walker, Lucie Riglet, Selin Zeyrek, Edwige Moyroud

**Affiliations:** ^1^ Sainsbury Laboratory University of Cambridge 47 Bateman Street Cambridge CB2 1LR UK; ^2^ Department of Genetics University of Cambridge Downing Street Cambridge CB2 3EH UK

**Keywords:** evo‐devo, flavonoid biosynthesis, *Hibiscus trionum*, MYB transcription factor, petal patterning, plant–pollinator interactions, replicated evolution

## Abstract

- Colorful petal patterns fulfill important functions and constitute excellent systems to illuminate the evolutionary processes that generate morphological diversity or instead support the repetitive emergence of similar forms.
- Here, we combined phylogenomic approaches, genetic manipulations, molecular techniques, and bee behavioral experiments to (i) solve the species relationships across the *Trionum* complex, a small *Hibiscus* clade that displays bullseye petal patterns varying in size, hue, and composition, (ii) identify key genes involved in the production of bullseye pigmentation, and (iii) reveal molecular events underpinning pattern variation during the evolution of the group.
- We found that epidermal cell shape, texture, and pigmentation are genetically distinct and that pigmentation is the most labile feature across the group. We demonstrate that repetitive bullseye reduction events primarily occur through independent modifications of a single genetic locus encoding BERRY1, an R2R3 MYB (myeloblastosis) that regulates anthocyanin pigment production in petals. We also found that buff‐tailed bumblebees discriminate against flowers with smaller bullseye sizes, suggesting that changing bullseye proportions impact plant–pollinator interactions.
- Our results demonstrate how repeated mutations in a single locus led to morphological variation in petal patterning, a trait shown to impact plant fitness in other species and contribute to angiosperm reproductive isolation and speciation.

## Introduction

The molecular basis underlying the repeated evolution of similar morphological characteristics across the tree of life remains a central question of evolutionary biology. Replicated evolution, a term encompassing both parallel and convergent evolution, leads different species and populations to independently evolve similar features (James *et al*., 2023). While this sometimes relies on the modification of homologous genes (Lenormand *et al*., 2016), it remains unclear why some genes are more likely than others to mediate parallel or convergent changes. The evolution of color patterns in both plants and animals provides phenotypically striking traits that can be utilized to unravel the molecular mechanisms supporting the evolution of diversity (Manceau *et al*., 2010; Orteu & Jiggins, 2020; Galipot *et al*., 2021). With > 350 000 extant species, the angiosperms are a living repository of natural diversity. The diversification of flowering plants yielded a remarkable array of morphologies and colorful patterns that decorate corollas. Remarkably, similar motifs are often found in distantly related species. Striking examples include petal stripes in *Gazania* and *Phlox*; venation patterns in *Veronica*, *Geranium*, and *Antirrhinum*; and bullseye patterns in the poached egg plant (*Limnathes douglasii*) and baby blue eyes (*Nemophila menziesii*). Such commonalities hint toward a limited but functionally important set of patterns that independently arose across distantly related angiosperms. It also provides multiple systems with which to investigate the processes that have led to the repeated emergence of similar traits.

Petal patterns play crucial roles in key biological processes including plant–pollinator communication, defense against herbivory, and protection from abiotic factors and thus are likely to contribute directly to plant fitness (Fairnie *et al*., 2022). Insect pollinators can display innate or acquired preferences for given motifs influencing their choice of flower to visit (Gumbert, 2000). Importantly, petal patterns also function postlanding, acting as visual guides to lure pollinators toward pollen and/or nectar rewards and increase the chances of successful pollination. The lower petal lobes of the purple monkeyflower (*Mimulus lewisii*), for example, display prominent yellow markings surrounded by dark pink stripes with a white border. Mutants lacking these nectar guides or with reduced petal coloration exhibit significant reductions in pollinator visitation (Owen & Bradshaw Jr., 2011). Further work in yellow monkeyflowers (*Mimulus guttatus*) reinforced the importance of pigmentation patterns for pollinator attraction as bumblebees preferred the flowers of the *red tongue* mutant with an expanded anthocyanin region over wild‐type flowers with smaller anthocyanin spots (Ding *et al*., 2020). Petal patterns also play important roles in protecting against abiotic stress. In wild sunflowers (*Helinathus annuus*), larger UV‐absorbing bullseyes increased a flower's tolerance to desiccation (Todesco *et al*., 2022). In silverweed flowers, the size of the UV bullseye positively correlates with the amount of exposure to UV irradiation, possibly enhancing pollen protection from UV‐induced DNA damage (Koski & Ashman, 2015). In both species, the UV‐absorbing bullseye results from the preferential or restricted production of flavonols at the petal base which protect against environmental stress (Pollastri & Tattini, 2011). Hence, the evolution of petal patterns is shaped by both biotic and abiotic factors, and investigating the processes mediating changes in petal patterning provides insights into the mechanisms facilitating the emergence of adaptation and speciation.

The patterns on the petal surface are produced by a combination of features including epidermal cells that differ in shape, texture, and pigmentation (Kay *et al*., 1981; Johnson & Midgley, 1997). Petal pigments are divided into three main classes: carotenoids, betalains, and flavonoids. The carotenoid and flavonoid biosynthetic pathways are widely distributed across the plant kingdom, while betalains are restricted to the order Caryophyllales. Flavonoids are the predominant class of pigments in angiosperm flowers and produce a wide array of colors. Flavonoids comprise several subgroups, including flavonols and anthocyanins, which are colorless/‘cream’‐like and blue/red, respectively. Flavonoid biosynthesis begins with the production of chalcone, followed by several hydroxylation steps to produce the precursors for flavonols and anthocyanins. Thereafter, the pathway branches, whereby the enzymes dihydroflavonol 4‐reductase (*DFR*) and anthocyanidin synthase (*ANS*) produce anthocyanins, while flavonol synthase (*FLS*) yields flavonols, respectively (Tanaka *et al*., 2008). The accumulation of these pigments, in varying combinations and abundance, and in different regions of the petals, gives rise to the spectrum of color patterns on the petal epidermis. Petal coloration can also be influenced by the physical properties of epidermal cells (Moyroud *et al*., 2017; Riglet *et al*., 2021). For instance, conical cells can focus light into the anthocyanin‐containing vacuole, enhancing color intensity, while semi‐ordered striations of the cuticle can produce a weak iridescence and blue halo effect that increases flower salience to pollinators (Whitney *et al*., 2009; Moyroud *et al*., 2017).

The genetic components underpinning the production of petal patterns have been functionally characterized in a limited number of model species. Flavonoid biosynthesis is regulated at the transcriptional level by the MBW (MYB‐bHLH‐WD repeat) complex, combining two types of transcription factors (TFs), an R2R3 MYB (myeloblastosis) and a basic helix–loop–helix (bHLH) protein, and a scaffold WD (tryptophan‐aspartate)‐repeat protein (Ramsey & Glover, 2005). MYBs and bHLHs constitute two of the largest TF families in plants, and as such, several MBW complexes exist to tightly regulate the expression of late flavonoid biosynthetic genes. In *Antirrhinum majus*, the venation pattern present on the lateral sides of the corolla tube is produced by overlapping expression patterns of the MYB TF AmVENOSA and its bHLH partner AmDELILA. Together, they combine with a WD40 co‐activator to form an MBW complex that activates anthocyanin biosynthesis (Shang *et al*., 2011). *AmVENOSA* expression is restricted to a wedge of cells between the vein and adaxial epidermis, while *AmDELILA* is broadly expressed in the epidermal cells of the corolla (Goodrich *et al*., 1992; Jackson *et al*., 1992). Therefore, the restricted expression of *AmVENOSA*, the MYB element of the MBW complex, is the main driver of the venation pattern. The mechanisms accounting for the spatial regulation of *MYB* transcription have only been explored in a few systems, including *Mimulus verbenaceus* leaf stripes (LaFountain *et al*., 2024) because many species used in the study of petal patterning cannot be genetically manipulated with ease. Hence, the upstream processes accounting for the restriction of *MYB* expression are still poorly understood across angiosperms.

Venice mallow (*Hibiscus trionum*) produces flowers with a striking bullseye pattern. This herbaceous species has emerged as a model system to understand petal pattern evolution and development at both the macro‐ and microscale (Moyroud *et al*., 2022; Riglet *et al*., 2024). Petal epidermal cells in the two regions of the bullseye exhibit differences in color, cell shape, and texture. Cells in the proximal region (petal base) are purple, flat, and elongated (tabular) and covered with a striated cuticle, while cells in the distal region (petal tip) are cream‐colored and conical in shape with a smooth surface (Vignolini *et al*., 2015). Differences in pigmentation between the two regions appear between stages 1 and 2 of floral development, while differences in cell shape and texture emerge later between stages 3 and 4 (Moyroud *et al*., 2022). The proximal and distal regions are specified very early during development, long before the epidermal cells acquire their characteristic features. During stage 0, a band of large elongated cells appears to mark the position of the future bullseye boundary and divide the petal epidermis into proximal and distal domains (Riglet *et al*., 2024). *Hibiscus trionum* belongs to the Malvaceae family and falls into the *Trionum* complex, which appears to have experienced a recent radiation event (Craven *et al*., 2011). The size of the bullseye varies extensively across the *Trionum* complex, providing an attractive system to investigate the processes underpinning petal pattern variation and possibly replicated evolution. In this study, we first used a phylogenomic approach to solve species relationships across the *Trionum* complex and uncovered multiple instances of bullseye pigmentation reduction. Combining molecular approaches and genetic manipulations *in planta*, we found that independent occurrences of bullseye size reductions are due to recurring modifications of an R2R3 MYB‐encoding locus, *BERRY1*. This locus restricts anthocyanin production to the petal base and acts as a principal driver of bullseye pattern formation. Importantly, pollinator behavior assays revealed that bumblebees easily discriminate between flowers of varying bullseye size, suggesting that repeated mutations within a single locus are sufficient to generate morphological variations in a trait likely to impact speciation.

## Materials and Methods

### Plant material and growing conditions

Wildtype *H. trionum* L. Cambridge University Botanic Gardens (CUBG) seeds (voucher CGE00046422) were sourced from the Cambridge University Botanic Gardens, Cambridge, UK. *H. trionum* L. seeds from Bream Head, New Zealand (vouchers AK253689 & CGE00046417) were sourced from Dr. Brian G. Murray (University of Auckland). *H. trionum* L. commercial seeds (voucher CGE00080883) were supplied by Doekele G. Stavenga (the Netherlands). Seeds of *H. richardsonii* Sweet ex Lindl. (vouchers AK251841 & CGE00046420) were also sourced from Dr. Brian G. Murray (University of Auckland). Seeds of *H. verdcourtii* Craven, population 1 (voucher CGE00046415), population 2 (voucher CGE00046414), population 3 (voucher CGE00080882), population 4 (voucher CGE00046413), population 5 (voucher CGE00046366), and *H. tridactylites* Lindl., population 6 (voucher CGE00046364) and population 7 (voucher CGE00046363), were provided by Stephen B. Johnson (New South Wales, Department of Primary Industries). Seeds were imbibed in 90°C H_2_O for 10 min and germinated in the dark at 30°C for 48 h. Seedlings were transferred to Levington High Nutrient M3 compost and grown under glasshouse conditions consisting of a 16 h : 8 h, light : dark photoperiod at 25°C with a minimum radiance of 88 W m^−2^. Wildtype species and transgenic lines used in this study are summarized in Tables S1 and S2, respectively.

### Phenotyping

Open flowers (stage 5, Moyroud *et al*., 2022) were collected in the morning and imaged on black velvet using a Panasonic DMC‐FZ38 camera. Dissected petals were imaged with a Keyence microscope (VHX5000 and VHX7000 models) fitted with a VH‐Z100R lens. Petals with the adaxial side facing upward were scanned with a black‐and‐white background using the Epson Perfection V850 Pro scanner. Total petal and purple pigmented areas were measured from scanned images using ImageJ v.1.53 (https://imagej.net/ij/). For each flower, three petals were measured and a minimum of 10 flowers were measured per genotype.

### Flavonoid extraction and quantification

Sample collection and extraction: stage 5 petals were dissected into proximal, boundary, and distal regions and flash frozen along with ovary tissue in liquid nitrogen. For each sample, *c*. 40 mg of tissue was homogenized in a tissue lyser. 1 ml of acidic methanol (70 : 30, MeOH : 1% acetic acid) was added to the tissue, vortexed, and then incubated overnight on a rotating wheel at 4°C. Petal debris was pelleted for 3 min at 13 148 ***g***, and the supernatant was collected and stored at 4°C until the following day. The extraction was repeated with 1 ml of acidic methanol (90 : 10, MeOH : 1% acetic acid). Supernatants from both extractions were pooled before reducing to *c. *250 μl by evaporation in a miVac centrifugal concentrator (Genevac, Ipswich, Suffolk, UK). The final volume was measured before measurements were taken.

Sample quantification: Flavonols and anthocyanins were quantified using a spectrophotometer set to A_350_ and A_530_, respectively. Three technical replicates (three dilutions) per extraction were measured. Concentrated extracts were diluted in 1 ml MeOH to measure flavonol concentration and in 1 ml 90 : 10, MeOH : 1 N HCl to measure anthocyanin concentration. Absorbance units were converted to concentration (mg flavonol or anthocyanin equivalent g^−1^ of fresh weight tissue) based on the following calculation:

$$\frac{\left(\frac{\left(\text{absorbance}\;\times \;\text{conversion factor}\;\times \;\text{volume total cuvette}\;\times \;\text{volume total extract}\right)}{\text{volume sample loaded}}\right)}{\text{mass of tissue}}$$

To determine conversion factors, standard curves were generated using the flavonol quercetin 3‐d‐galactoside (item 18648; Cayman Chemicals, Ann Arbor, MI, USA) using the anthocyanin cyanidin 3‐O‐glucoside (chloride) (item 16406; Cayman Chemicals) as reference compounds.

### Crosses

For each cross, pollen was transferred by hand to the recipient flower. To avoid self‐fertilization, recipient flowers without visible pollen on their stigma were selected for crossing.

*H. trionum* CUBG × *H. richardsonii*: five independent crosses between *H. trionum* CUBG and *H. richardsonii* were performed. F1 hybrids were identified based on the presence of a pigmented bullseye and microscopy imaging. F1 hybrid plants were allowed to self‐pollinate, and the flower phenotypes of the next‐generation (F2) individuals were characterized.

*H. verdcourtii* pop. 2 × *H. verdcourtii* pop. 3: pollen was transferred by hand from a *H. verdcourtii* flower (St. George, red bullseye, ‘*H. ver2*’) to an open *H. verdcourtii* flower (St. George, pale yellow with pink halo bullseye, ‘*H. ver3*’). F1 hybrid plants were allowed to self‐pollinate, and flower phenotypes of the F2 generation were characterized.

### Rapid genomic DNA extraction to isolate *HrBERRY1*

Fresh leaf tissue (*c*. 1 cm^2^) was ground with a plastic pestle before the addition of 250 μl Tris‐HCl‐EDTA extraction buffer with 10% SDS. The samples were briefly vortexed and spun for 1 min at 13 148 ***g*** to collect genomic DNA from the supernatant. DNA was precipitated with isopropanol, and the pellet was washed with 70% EtOH. The pellet was dried before resuspension in 40 μl ddH_2_O.

### Genotyping the F2 progeny from wild‐type *H. trionum* and *H. richardsonii* crossing

A single bud per individual was collected from *H. trionum* CUBG and *H. richardsonii* parents, and from 27 F2 individuals randomly chosen among > 100 F2 individuals that had been phenotyped and assigned to one of three phenotypes (*H. trionum* CUBG‐like, *H. richardsonii*‐like, hybrid‐like). Genomic DNA was extracted from selected individuals. *C*. 800 mg of mixed petal/bud tissue was divided into 50 mg aliquots, homogenized, and incubated with 1 ml cetyltrimethylammonium bromide (CTAB) buffer (2% w/v) + 10 μl 2‐mercaptoethanol (M3148; Sigma) at 55°C for 30 min. The supernatant was collected from pelleted samples and incubated with 10 mg ml^−1^ RNase A (EN0531; ThermoFisher Scientific, Waltham, MA, USA) at 32°C for 30 min. DNA was extracted using 25 : 24 : 1, phenol : chloroform : isoamyl alcohol saturated with 10 mM Tris, pH 8.0, 1 mM EDTA (P0269; Sigma) and 24 : 1, chloroform : isoamyl alcohol mixture (25666; Sigma). DNA was separated from protein and other cellular debris by carefully removing the upper aqueous layer after each extraction. The DNA was precipitated overnight at −20°C in chilled isopropanol and washed twice with 70% EtOH. DNA pellets were dried and resuspended in 30 μl low Tris EDTA (TE) buffer (10 mM Tris HCl pH 8, 0.1 mM EDTA, pH 8.0). Genomic DNA purity and concentration were analyzed using a NanoDrop (ThermoFisher Scientific). *HtBERRY1* or *HrBERRY1* gene copies were determined by PCR using allele‐specific primers (Table S3). A 1200‐bp band indicated the presence of the *HtBERRY1* allele and a 400‐bp band indicated the presence of the *HrBERRY1* allele. Samples were genotyped in a blind manner. To confirm the genotyping results, a further 11 F2 individuals with a *H. richardsonii*‐like phenotype were tested. *HtACTIN1* was included as a positive control for the presence of genomic DNA.

### Genotyping the F2 progeny from *H. ver2* and *H. ver3* crossing

Fresh leaf tissue (*c*. 1 cm^2^) was collected from *H. ver2* and *H. ver3* parents and 33 F2 individuals. The tissue was ground with a plastic pestle before the addition of 250 μl Tris HCl‐EDTA extraction buffer with 10% SDS. The samples were briefly vortexed and spun for 1 min at 13 148 *g* to collect the genomic DNA from the supernatant. DNA was precipitated with isopropanol, and the pellet was washed with 70% EtOH. The pellet was dried before resuspension in 40 μl ddH_2_O. Samples were genotyped in a blind manner to check for the presence of *HvBERRY1*. The presence of *HvBERRY1* was determined by PCR using *HvBERRY1*‐specific primers (Table S3). F2 samples with a pigmented red bullseye produced a 421‐bp band that indicated the presence of *HvBERRY1*, while those without a pigmented bullseye did not produce a PCR product. *HvACTIN1* was included as a positive control for the presence of genomic DNA.

### Tissue collection, RNA extraction, and Illumina sequencing to produce the mixed tissue transcriptome for phylogenomic analyses

Whole buds, flowers, and fruit tissues were collected from each of the following species and populations: wild‐type *H. trionum*, *H. trionum* diploid New Zealand naturalized race, *H. trionum* Commercial, *H. richardsonii*, *H. verdcourtii* pops. 1–5, *H. tridactylites* pops. 6 & 7. Upon collection, bud tissue was flash frozen in liquid nitrogen. Per species/population, three biological replicates were collected, with each biological replicate corresponding to one individual plant. Frozen samples were homogenized using a pestle and mortar, and total RNA was extracted using the Spectrum Plant Total RNA Kit (STRN250; Sigma). *C*. 40 mg of tissue was used for each RNA extraction. An on‐column DNase I treatment (Sigma DNASE70) was performed between the first two column washes. RNA was eluted in 30 μl nuclease‐free H_2_O (MC1191; Promega), and purity was analyzed using a NanoDrop (ThermoFisher Scientific) and RNA ScreenTape (Agilent 5067–5576, 5067–5577, 5067–5578) on the Agilent TapeStation 2200 system.

Samples with a minimum of 1.7 μg total RNA (> 50 ng μl^−1^) and an A_260/280_ value of 2.0 were shipped on dry ice to Azenta Life Sciences, Leipzig (formerly GeneWiz) for RNA‐seq library preparation and sequencing. Library preparation was performed with polyA selection and sequencing using an Illumina HiSeq machine (2×150 bp configuration, *c*. 350 M raw paired‐end reads per lane, at least 30 M reads per sample). Raw reads (accession nos. SAMN47801648 to SAMN47801669) have been deposited on GenBank SRA (https://www.ncbi.nlm.nih.gov/sra).

### Phylogenomic transcriptome analysis

Raw sequence reads for the outgroups (*Hibiscus cannabinus*, *Hibiscus syriacus*, and *Hibiscus sabdariffa*) were downloaded from the NCBI Short Read Archive (SRA) accessions (SRX1084555 (Zhang *et al*., 2015), SRX2142897 (Kim *et al*., 2017), SRX3341155 (Loo *et al*., 2016)). The sequences were assembled using the same procedure as the *H. trionum* stage 1 petal transcriptome (described below).

We reduced the redundancy for each transcriptome using cd‐hit‐est v.4.7 (Fu *et al*., 2012), with the settings ‘‐c 0.99 ‐n 10 ‐r 0’. Initial homology inference was conducted by performing an all‐by‐all Blastn v.2.7.1 with the settings ‘‐‐e‐value 10 –max_target_seqs 1000’. Markov clustering, as implemented in the program mcl (van Dongen, 2000) with an inflation value of 1.4, was used to refine the clusters further. We then performed two rounds of phylogeny‐based data cleaning. In the first round, the sequences were aligned using Mafft v.7.407 (Katoh & Standley, 2013) with the settings ‘—auto –maxiterate 1000’; the alignments were cleaned for 10% column occupancy using the program pxclsq with the settings ‘‐p 0.1’ from the phyx package (Brown *et al*., 2017). A tree for each cleaned alignment was inferred using maximum likelihood as implemented in FastTree v.2.1.1 (Price *et al*., 2010) with the settings ‘‐gtr ‐nt’. The resulting trees had any tips with an absolute branch length of 0.4 subs/site or a relative branch length of 0.6 subs/site removed. Only the transcript with the most informative sites was retained for any instance where the same taxa were sister to one another. Any subclades containing at least four taxa and were on a branch with a 1.0 subs/site length were divided into separate homolog trees. During the second round of cleaning, the same process was performed; however, the maximum likelihood tree inference was conducted using IQtree v.1.6.12 (Nguyen *et al*., 2015) using the best‐inferred model of evolution (Kalyaanamoorthy *et al*., 2017) and 1000 ultrafast bootstrap replicated. The orthologs were extracted from the trees using the maximum Inclusion approach with an absolute branch length setting of 0.4 subs/site and a relative branch length setting of 0.6 subs/site. A coalescent‐based maximum quartet support species tree was estimated using Astral v.5.7.3.

### Tissue collection, RNA extraction, and Illumina sequencing for *H. trionum* stage 1 petal transcriptome

Tissue was collected from five individual *H. trionum* CUBG plants to generate five biological replicates: for each biological replicate, stage 1 petals were harvested and dissected to separate proximal and distal regions. Tissues from these 10 samples were flash frozen in liquid nitrogen, ground to a fine powder, and total RNA extracted using the Spectrum Plant Total RNA kit (STRN250; Sigma) with *c.* 40 mg of starting material. An on‐column DNase I (DNASE70; Sigma) treatment was performed between the first two column washes. RNA was eluted with 30 μl nuclease‐free H_2_O (MC1191; Promega). RNA purity was analyzed using a NanoDrop (ThermoFisher Scientific) and RNA ScreenTape (Agilent 5067–5576, 5067–5577, 5067–5578) on the Agilent TapeStation 2200 system. Samples with a minimum 1.75 μg total RNA (> 50 ng μl^−1^) and A_260/280_ value of 2.0 were shipped on dry ice to Azenta Life Sciences, Leipzig (formerly GeneWiz) for RNA‐seq library preparation and sequencing. Library preparation was performed with polyA selection and sequencing using an Illumina HiSeq machine (2×150 bp configuration, *c*. 350 M raw paired‐end reads per lane, at least 30 M reads per sample). Raw reads (accession nos. SAMN47793371 to SAMN47793390) have been deposited on GenBank SRA (https://www.ncbi.nlm.nih.gov/sra).

### *Hibiscus trionum* CUBG stage 1 petal transcriptome assembly and differential gene expression analysis

RNAseq reads were used to assess gene expression and compare transcript abundance between proximal and distal regions of stage 1 (S1) petal primordia, as defined in Moyroud *et al*., 2022. First, a reference transcriptome (Dataset S1) was created by combining the raw reads from S1 proximal and distal petal tissue of a single plant. To produce this reference transcriptome, the raw reads were cleaned, assembled into a database with chimeric sequences removed, and their coding regions for transcripts predicted using the previously excluded chimeric sequences. Cleaning raw reads used Rcorrector v.1.0.3 (Song & Florea, 2015) to remove sequencing errors, and Trimmomatic v.0.38 (Bolger *et al*., 2014) to filter for quality and remove the adapters added during library preparation. The cleaned reads were then assembled into a transcriptome with Trinity v.2.8.3 (Grabherr *et al*., 2011). The procedure followed Yang & Smith (2013) with a database composed of the genomes of *Gossypium raimondii*, *Arabidopsis thaliana*, and *Theobroma cacao*. Chimeric sequences were filtered by Blast® v.2.2.31 (Altschul *et al*., 1997) and isoforms and assembly artifacts were removed using Corset v.1.07 (Davidson & Oshlack, 2014). Finally, coding regions were predicted using the program Transdecoder v.5.3.0 (Haas *et al*., 2013) and a Blast database composed of the same taxa as the chimera database.

The reference transcriptome was then used as a guide for transcriptome assembly corresponding to each biological replicate. Raw reads from each sample were first mapped onto the reference transcriptome using Kallisto v.0.44.0 (Bray *et al*., 2016). The transcript‐level abundance was converted into counts using the program tximport (Soneson *et al*., 2015). Fold change and dispersion of these counts were calculated using DESeq2 (Love *et al*., 2014). Finally, the top blastn hit between the transcriptome sequences and the NCBI nr database was used for automatic gene annotation. Genes with log_2_‐fold change ≤ 2 or > 2 and adjusted *P*‐value < 10^−5^ were considered differentially expressed. Results of the differential gene expression analysis and assembled transcripts from stage 1 *H. trionum* CUBG petals are provided in Dataset S1 and S2.

### Phylogenetic analysis to establish the identity of selected R2R3 MYBs

The amino acid sequences of HtBERRY1 (HRI_002225000), HtBERRY2 (HRI_002225200), and HtCREAM1 (HRI_001263100) were used to conduct a Blast search against the genome of *H. trionum* available on GenBank (https://www.ncbi.nlm.nih.gov/datasets/genome/GCA_030270665.1/). This yielded another six *HtBERRY*‐like sequences (HRI_00224000, HRI_002224800, HRI_002224600, HRI_000181400, HRI_001579800, and HRI_002131000) and another three *HtCREAM*‐like sequences (HRI_003566500, HRI_00625700, and HRI_001075200). Those 12 *H. trionum* sequences were combined with protein sequences of subgroup six MYB genes known to regulate anthocyanin production in other species: ROSEA1 (DQ275529), ROSEA2 (DQ275530) and VENOSA (DQ275531) from *A. majus*; PELAN (KJ011144) and NEGAN (KJ011145) from *M. lewisii*; MYB113‐like (XM_012977147) from *M. guttatus*; DEEP PURPLE (HQ116169) and PURPLE HAZE (HQ116170, HQ428100) from *Petunia hybrida*; also amino acid sequences of all MYB genes identified in the genome of *A. thaliana*, *G. raimondii*, and *T. cacao* were acquired from Jiang & Rao (2020), He *et al*. (2016), and Du *et al*. (2022), respectively. In total, 483 MYB sequences were used for phylogenetic reconstructions, and those are provided in Dataset S3. The 483 sequences were aligned using Mafft v.7.490 with the setting ‘—auto –maxiterate 1000’. The alignment was then cleaned for a minimum of 10% column occupancy using the program pxclsq from the phyx package. A maximum likelihood tree was then inferred from the cleaned alignments using the WAG model of evolution, with gamma rate variation and 1000 ultrafast bootstrap replicates as support (Hoang *et al*., 2018).

### cDNA synthesis

cDNA was synthesized from 0.5 μg total RNA using SuperScript III Reverse Transcriptase (Invitrogen). Oligo(dT)_15_ primers were used for first‐strand cDNA synthesis for gene isolation and measuring expression by reverse transcription polymerase chain reaction (RT‐PCR) or quantitative polymerase chain reaction (qPCR). First‐strand cDNA synthesis followed the manufacturer's guidelines, with incubation with SuperScript III RT at 50°C for 1 h followed by an inactivation step at 70°C for 15 min.

### Construction of plant expression vectors

The coding sequences of *HtDFR1*, *HtBERRY1*, and *HtCREAM1* were amplified by PCR using Q5 High‐Fidelity DNA polymerase with primers listed in Table S3. Each PCR product was cloned into the EcoRV‐digested pBluescript KS(‐) vector. Restriction enzyme digestion was used to extract the coding sequence of each gene and insert it into a modified pGREENII (for *HtDFR1* and *HtCREAM1* overexpression (OE)) or pCAMBIA (for *HtBERRY1* OE) containing a 2x35S CaMV promoter driving transgene expression. The plant expression vectors also express a fluorescent eYFP under the control of the promoter region of *AtUBIQUITIN10* (PromUBQ10). Final vectors were sent to Azenta (formerly Genewiz) for Sanger sequencing to validate the final construct before plant transformation. The final vectors used for plant transformation are summarized in Table S4.

### Plant transformation

Electrocompetent *Agrobacterium tumefaciens* LBA4404 cells were transformed with plant expression vectors pVT9 (*2x35S::HtDFR1*), pAF29 (*2x35S::HtCREAM1*), or pAF52 (*2x35S::HtBERRY1*). Transformed cells were selected on LB agar plates containing 50 mg l^−1^ kanamycin and 25 mg l^−1^ streptomycin after 48 h incubation in the dark at 30°C. Transgenic *H. trionum* lines were produced using the protocol described in Moyroud *et al*., 2022 with the following modification: 100 mg l^−1^ of carbenicillin was used instead of 250 mg l^−1^ of cefotaxime for the MS Hib plates.

### Gene expression analysis using quantitative RT‐PCR

To quantify gene expression levels during petal development in *H. trionum* CUBG and *H. richardsonii*, petals from stages 1–4 were dissected into proximal and distal regions. Stage 3 petals were dissected into proximal and distal regions to measure expression in the transgenic lines of *35S::HtDFR1* overexpression (OE) and knockdown (KD), *35S::HtCREAM1*, and *35S::HtBERRY1*. Three biological replicates were collected from individual plants for each stage. Frozen petal tissue was ground to a fine powder, and total RNA was extracted using the Spectrum Plant Total RNA kit (STRN250; Sigma) with *c*. 40 mg of ground tissue used as starting material for each RNA extraction. cDNA was synthesized from total RNA using SuperScript III Reverse Transcriptase (Invitrogen) following the manufacturer's guidelines. Quantitative real‐time PCR was performed using a Roche LightCycler 480 machine and the Luna Universal qPCR Master Mix (New England BioLabs, Ipswich, MA, USA). *HtACTIN1* was used as a housekeeping gene for all biological replicates and stages, as in Moyroud *et al*. (2022). Data were analyzed using a modified delta Ct method, considering the real efficiency of each primer pair and one reference gene for normalization (Pfaffl, 2001; Ganger *et al*., 2017). Statistical analyses were calculated in RStudio (v.1.1.1717). A Student's *t*‐test, Welch's *t*‐test, or Wilcoxon test was used depending on the normality and homogeneity of variance of the samples.

### Bumblebee behavior assay

Experiments with flower‐naïve buff‐tailed bumblebees (*Bombus terrestris* v. audax; Research Hive, BioBest UK) were conducted using the design published in Moyroud *et al*. (2017). Each colony was fed daily with fresh 15% sucrose solution and twice a week with pollen grains (The Happy Health Co., Brighton, UK). Foragers were hand‐marked with water‐based Thorne queen marking paints in various colors to distinguish them during the experiments. For the preference tests, one *H. trionum* CUBG and one *H. richardsonii* open flower were each placed in a tube containing water equidistant from the hive entrance. Per test, one naïve bumblebee was released from the hive into the arena and the flower it chose to land on first (preference) was recorded. Forty individual bumblebees were tested for their flower preference. Statistical differences were calculated using a one‐sample *t*‐test in RStudio (v.1.1.1717).

## Results

### Bullseye pattern reduction occurred several times independently across the *Trionum* complex

**Fig. 1:**
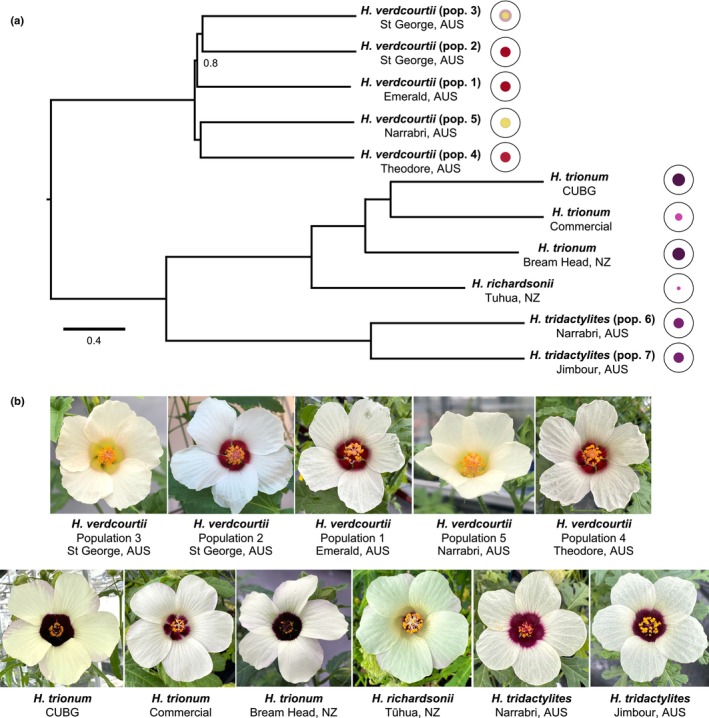
Phylogenetic relationships between the *Hibiscus* species of the *Trionum* complex. (a) Phylogenetic tree highlighting relationships between species and populations. Unless stated, all nodes are perfectly supported. Branch lengths are shown in coalescent units. Each species is represented by a cartoon depicting bullseye size and color. (b) Representative flowers of each species/population emphasizing variation in bullseye size and color.

Analyzing trait distribution across a species tree often represents a first step toward understanding how these traits vary over time and how these changes relate to the evolutionary history of a specific group of organisms. The indigenous or naturalized status of several species in the *Trionum* complex has been debated, and relationships within the group have remained obscure and unresolved (de Lange, 2008; Craven *et al*., 2011). Thus, to investigate petal pattern evolution across the *Trionum* complex, we first generated transcriptomic data using RNA sequencing and undertook a phylogenomic approach to clarify the relationships among the four species within the group (Fig. 1a, S1). Whenever possible, we included several accessions from each species to capture the morphological diversity existing within some species (Fig. 1b; Craven *et al*., 2011; Moyroud *et al*., 2022). For instance, *H. verdcourtii* individuals from Emerald and Theodore (populations 1 and 4, respectively; Fig. S1) exhibit a red bullseye, while Narrabri individuals (population 5; Fig. S1) produce cream‐colored flowers lacking a pigmented bullseye (Fig. 1b). By contrast, individuals with different bullseye patterns can be found in the same region. For example, plants with red bullseye flowers (*H. verdcourtii* population 2; Fig. S1) or cream flowers with a faint pink halo (*H. verdcourtii* population 3; Fig. S1) are both present in St. George (Craven *et al*., 2011; Figs 1b, S1). Intraspecies variation also exists within *H. trionum*, whereby a commercial accession produces a visibly smaller bullseye than our wild‐type reference accession *H. trionum* CUBG (Moyroud *et al*., 2022; Riglet *et al*., 2024) and wild individuals from Bream Head, New Zealand (Figs 1b, S1; Craven *et al*., 2011; Moyroud *et al*., 2022).

As expected, accessions assigned to the same species clustered together in our phylogenomic reconstruction (Fig. 1a). Our analysis also identified *H. richardsonii*, the other diploid species of the group (de Lange, 2008; Murray *et al*., 2008), as sister to *H. trionum*. We found that the three main features of petal epidermal cells (pigmentation, shape, and texture) can vary independently from each other. All accessions, regardless of their corolla pigmentation pattern, produced smooth conical cells in the distal petal region (petal tip) and flat elongated (tabular) striated cells in the proximal petal domain (petal base) except for the five *H. verdcourtii* accessions that all exhibit smooth tabular cells in the proximal region (Fig. S2). Finally, our phylogenomic reconstruction identified pigmentation to be a more labile trait than the structural features (shape and texture) of epidermal petal cells, as accessions lacking pigmentation at the petal base (*H. verdcourtii* populations 3 and 5) or producing a reduced pigmented bullseye (*H. trionum* Commercial and *H. richardsonii*) did not cluster together (Fig. 1a). The multiple instances of bullseye pigmentation reduction across the *Trionum* complex therefore provide an excellent opportunity to study the genetic basis of replicated evolution.

### A single genetic locus accounts for the reduction of the pigmented bullseye in *H. richardsonii*, the sister species of *H. trionum*

**Fig. 2:**
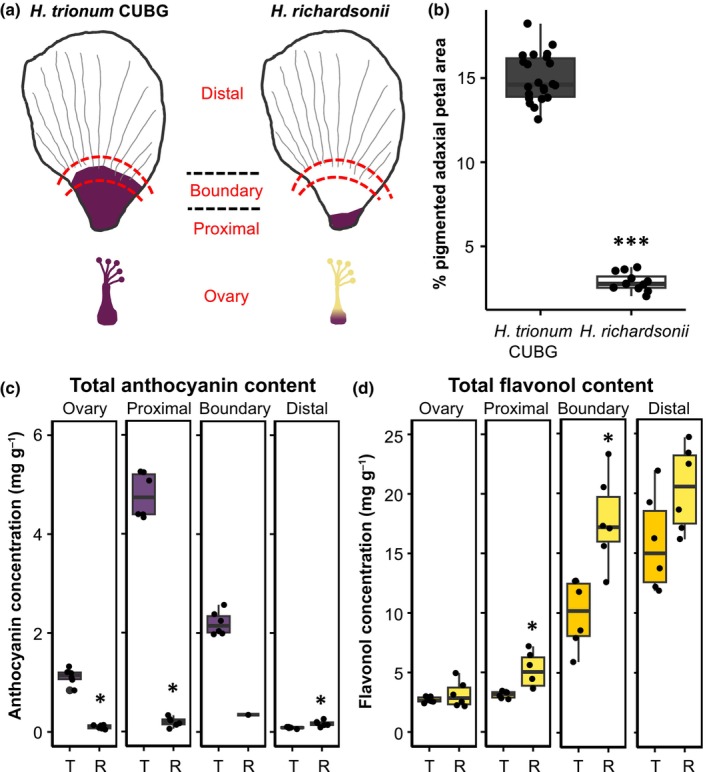
Morphological differences in petal patterning between *Hibiscus trionum* Cambridge University Botanic Gardens (CUBG) and *Hibiscus richardsonii*. (a) Schematic depicting petal regions and ovary of a stage 5 flower. For pigment extraction assays, petals were dissected into ovary, proximal (tabular striated cells), boundary (tabular smooth cells), and distal (conical smooth cells) regions, demarcated by the red dashed lines. (b) The size of the pigmented bullseye was measured as a percentage of the pigmented region over total petal area. *n* = 22 and *n* = 12 flowers for *H. trionum* CUBG and *H. richardsonii*, respectively. Three petals were measured per flower. ***, *P* < 0.001 (Student's *t*‐test). (c) Anthocyanin and (d) flavonol quantification of *H. trionum* CUBG (T) and *H. richardsonii* (R) flowers. Stage 5 flowers were dissected into four regions as highlighted in (a). Six flowers were analyzed per species. Each data point represents the average of three technical replicates. *, *P* < 0.05 (Welch's or Student's *t*‐test) of pairwise comparisons made between species in the same region.

We previously found that the bullseye boundary separating proximal and distal regions is specified closer to the petal base during the pre‐patterning phase in *H. richardsonii* compared to *H. trionum* CUBG (Riglet *et al*., 2024). This boundary shift is sufficient to produce a smaller structural bullseye in *H. richardsonii* as striated tabular cells cover a smaller portion of its petal. However, the pigmented area appears even smaller than the structural bullseye (Fig. 2a) suggesting that additional changes affecting the control of pigment production rather than the pre‐patterning process itself must also have occurred during evolution along the lineage leading to *H. richardsonii* (Riglet *et al*., 2024). In this study, we aim to clarify the genetic basis accounting for this reduction in pigmentation.

To precisely characterize differences in pigmentation between *H. trionum* CUBG and *H. richardsonii* bullseyes, we examined and compared the morphological and chemical features of open flowers (stage 5) from both species. We found that the pigmented bullseye covers 15% of the petal surface in *H. trionum* CUBG, while the *H. richardsonii* bullseye represents only 3% of the petal surface (Fig. 2b). This fivefold reduction in pigmented bullseye size coincides with a 15‐fold reduction in anthocyanin levels in the *H. richardsonii* proximal region compared to *H. trionum* CUBG (Fig. 2c). The lower amount of anthocyanin recorded in *H. richardsonii* petals could be due to reduced anthocyanin production or increased anthocyanin degradation. We noticed that throughout bud development, *H. richardsonii* petal primordia always displayed a smaller pigmented area compared to *H. trionum* CUBG petals at equivalent stages (Fig. S3). This suggests differences in anthocyanin synthesis, rather than degradation, account for differences in bullseye sizes between the two species. Total flavonol content was 1.4‐ to 2.6‐fold higher in *H. richardsonii* petal tissue compared to *H. trionum* CUBG (Fig. 2d). The reduction in anthocyanin production in *H. richardsonii* could favor precursor availability toward the flavonol branch of the flavonoid pathway, accounting for the increase in flavonols recorded in the proximal and boundary regions of *H. richardsonii* petals (Fig. 2d). Taken together, our data indicate the pigmented bullseye of *H. trionum* CUBG is generated by restricting anthocyanin synthesis to the proximal petal, while flavonols are preferentially produced in the distal petal and that the smaller size of the pigmented bullseye in *H. richardsonii* is likely due to reduced anthocyanin synthesis.

**Fig. 3:**
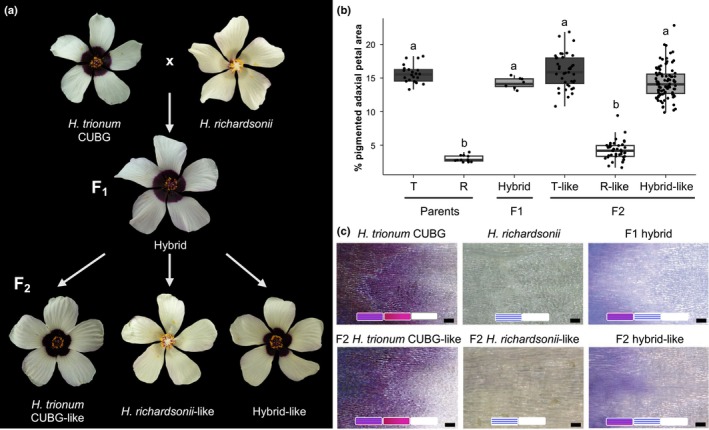
Characterization of *Hibiscus trionum* CUBG, *Hibiscus richardsonii*, F1 and F2 phenotypes. (a) F1 and F2 flower phenotypes of *H. trionum* CUBG crossed with *H. richardsonii*. The F1 hybrid produced flowers with a pigmented bullseye. The F2 selfing population yielded three flower phenotypes that recapitulate the two parental and F1 hybrid flower morphologies. (b) Measurement of the pigmented bullseye size reflects F2 phenotypes that match parental (*H. trionum* CUBG = T, *H. richardsonii* = R) and F1 hybrid phenotypes. Different letters indicate statistically significant differences (Tukey's HSD, *P* < 0.0004). (c) Imaging of the boundary region of petals from the six flower morphologies from (a). The bullseye boundary region is characterized by the presence of smooth, pigmented, tabular cells in *H. trionum* CUBG and F2 *H. trionum* CUBG‐like. Such cells are lacking in the bullseye boundary region *of H. richardsonii*, and F2 *H. richardsonii*‐like and striated, unpigmented, tabular cells are present instead. The bullseye boundary region of both F1 and F2 hybrid‐like flowers also lack smooth, pigmented, tabular cells but contain both pigmented and nonpigmented striated cells. Cell types present are summarized by symbols at the bottom of each microscopy image: striated, pigmented, tabular cells ‐ striped purple rectangle; smooth, pigmented, tabular cells ‐ solid purple rectangle; striated, nonpigmented, tabular cells ‐ striped white rectangle; smooth, nonpigmented, tabular cells ‐ solid white rectangle. Bars, 100 μm. Magnified images illustrating each cell type are presented in Supporting Information Fig. S4.

To start uncovering the molecular mechanisms accounting for the difference in bullseye pigmentation between *H. trionum* CUBG and *H. richardsonii*, we crossed the two species together (Fig. 3a). Hybrid flowers displayed a pigmented bullseye similar in size to *H. trionum* CUBG (Fig. 3a,b). Closer inspection of the boundary region (Fig. 3c) revealed differences in the composition and pattern of cell types between the hybrid and the *H. trionum* parent. In the parental *H. trionum* CUBG, the boundary region was characterized by three cell types: (1) striated, pigmented, tabular cells, (2) smooth, pigmented, tabular cells, and (3) smooth, nonpigmented, tabular cells (Figs. 3c, S4). In *H. richardsonii*, the reduction in bullseye pigmentation resulted in only two cell types at the boundary: (1) striated and (2) smooth, nonpigmented, tabular cells (Figs 3c, S4). Interestingly, in the hybrid, striated, nonpigmented, tabular cells replaced the smooth, pigmented, tabular cells found in *H. trionum* CUBG (Figs 3c, S4). Although the hybrid appears identical to *H. trionum* CUBG at the macroscale, the hybrid differs in boundary composition. In the F2 selfing population, the two parental and hybrid phenotypes were recapitulated, and these morphologies segregated in a Mendelian ratio as 40, 41, and 96 individuals displayed a *H. trionum* CUBG‐like, *H. richardsonii*‐like, and hybrid‐like phenotype, respectively. This indicates that a single locus likely accounts for differences in pigmented bullseye dimensions.

### Expression patterns of a *DFR* and a *FLS* *Hibiscus* homolog correlate with petal pigmentation

**Fig. 4:**
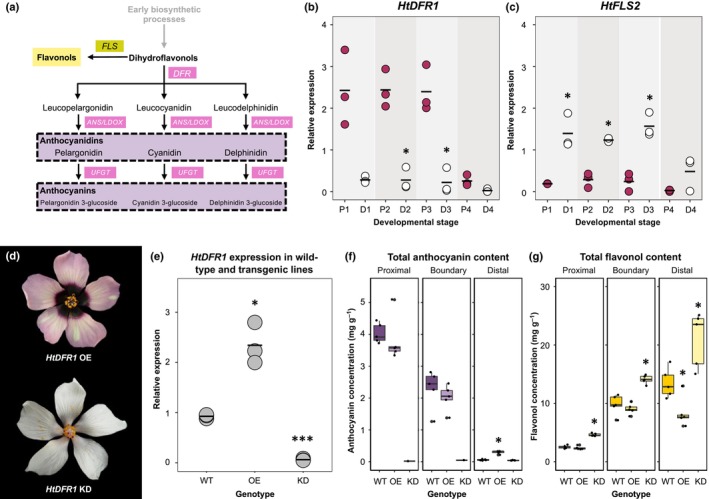
Bullseye pigmentation reflects the patterning of dihydroflavonol 4‐reductase (*DFR*) and flavonol synthase (*FLS*) expression across the petal primordia in *Hibiscus trionum* Cambridge University Botanic Gardens (CUBG). (a) Simplified flavonoid biosynthesis pathway highlighting DFRs as a shared common precursor for flavonol and anthocyanin production via *FLS* and *DFR* enzyme activity, respectively. Expression of (b) *HtDFR1* and (c) *HtFLS2* throughout development in proximal (P1–P4) and distal (D1–D4) petal tissue of *H. trionum* CUBG. *, *P* < 0.05 (Student's or Welch's *t*‐test) of pairwise comparisons made between proximal and distal tissue of the same stage. (d) Phenotypes of two *35S::HtDFR1* lines. Representative images of other independent transgenic lines are provided in Supporting Information Fig. S7. (e) Quantification of *HtDFR1* expression in distal tissue of *35S::HtDFR1* OE (overexpression) and proximal tissue of KD (knockdown) lines. *, *P* < 0.05; ***, *P* < 0.01 (Student's or Welch's *t*‐test) for comparisons of *HtDFR1* against equivalent petal region in WT. The expression of *HtBERRY1* in the proximal tissue of WT is given on the left for reference. The expression of *HtBERRY1* in the distal tissue of WT is extremely low (*c*. 0.009 average relative expression compared to *HtACTIN1* and thus is not represented on this plot). (f) Anthocyanin and (g) flavonol quantification in both types of *35S::HtDFR1* lines. Stage 5 petals were dissected into three regions as highlighted in Fig. 2(a). Five flowers were analyzed per line. Each data point represents the average of three technical replicates. *, *P* < 0.05 (Student's, Welch's *t*‐test, or Wilcoxon's test) of pairwise comparisons made between *HtDFR1* OE vs WT and *HtDFR1* KD vs WT tissue of the same region. Comparisons were not made in regions that had fewer than three data points as some replicates yielded non‐detectable amounts of anthocyanin. For expression data in (b), (c), and (e), three biological replicates were extracted per stage and each data point indicates an average of three technical replicates; horizontal bars indicate mean relative expression values. Expression values are relative to *HtACTIN1* expression levels. ANS, anthocyanidin synthase; LDOX, leucoanthocyanidin dioxygenase; UFGT, UDP‐glucose flavonoid glucosyltransferase.

Flavonoid biosynthesis is often targeted by evolutionary processes to generate diversity across flowering species (Kellenberger & Glover, 2023). We hypothesized that differences in petal patterning between *H. trionum* CUBG and *H. richardsonii* are also a consequence of modifications to the flavonoid pathway. This biosynthetic pathway is well‐characterized (Fig. 4a) and includes the production of a common dihydroflavonol precursor before splitting into two main branches that lead to anthocyanin or flavonol synthesis. DFR and FLS are key enzymes involved in the production of anthocyanins and flavonols, respectively. From the *H. trionum* genome, we isolated three homologs of each gene. We measured their expression in the proximal and distal regions of developing *H. trionum* CUBG petals (Figs 4b,c, S5). Among the three *DFR* homologs, *HtDFR1* was expressed at the highest levels in the proximal region: *HtDFR1* expression was 8‐ to 11‐fold higher at the petal base compared to the distal region throughout development (Fig. 4b). *HtDFR2* and *HtDFR3* expression levels in the proximal petal were almost negligible, and we concluded that they are not likely involved in anthocyanin production at the petal base (Figs S5, S6). *HtFLS2* was highly expressed during the first three stages of petal development (Fig. 4c) before declining at stage 4, following a similar temporal dynamic yet complementary spatial expression pattern to *HtDFR1* as its expression was 4‐ to 19‐fold higher in the distal region (Fig. 4c). Neither *HtFLS1* nor *HtFLS3* were expressed at levels comparable to *HtFLS2* (Figs S5, S6). These results demonstrate that *HtDFR1* and *HtFLS2* are likely the main players in shaping the bullseye pattern and the spatial accumulation of anthocyanins and flavonols (Fig. 2c,d) directly reflects their expression patterns.

To determine whether *HtDFR1* was responsible for bullseye anthocyanin production, we generated *35S::HtDFR1* transgenic lines in *H. trionum* CUBG. Four strong overexpression (OE) lines with ectopic pigmentation in the distal petal were recovered along with six transgenic lines that produced flowers devoid of a pigmented bullseye (Figs 4d, S7). We hypothesized this was due to *HtDFR1* silencing triggered by transcript overaccumulation as seen in *Arabidopsis* T‐DNA supertransformants (Schubert *et al*., 2004). To test this hypothesis, we quantified *HtDFR1* transcription levels in transgenic lines exhibiting both phenotypes. We found the presence of distal pigmentation was accompanied by a 300‐fold increase in *HtDFR1* expression in the distal region (Figs 4e, S7). Conversely, *HtDFR1* transcript levels were very low (15‐fold lower than WT expression) in the proximal petals of white flowers, confirming that *HtDFR1* expression correlated with the strength of the phenotype observed. We concluded the absence of pigmentation was due to *HtDFR1* silencing such that the transgenic lines producing white flowers corresponded to *HtDFR1* knockdown (KD). Total anthocyanin and flavonol measurements in both lines confirmed visual observations. Anthocyanin levels were fivefold higher in the distal petal of the *HtDFR1* OE line and almost absent from petals of the *HtDFR1* KD line (Fig. 4f). Flavonol levels were twofold higher throughout the petal of the *HtDFR1* KD line (Fig. 4g). The lack of *HtDFR1* activity in the KD lines likely increased precursor availability for flavonol biosynthesis, possibly accounting for increased flavonol levels.

Although *HtDFR1* OE produced distal petal pigmentation, anthocyanin levels remained lower in the distal petal than in the proximal region, suggesting that other factors, such as substrate competition between ectopic *HtDFR1* and endogenous *HtFLS2*, limit anthocyanin production in the distal petal. A clear bullseye pattern was still visible in the *HtDFR1* KD line despite the near‐complete absence of anthocyanin (Figs 4d, S7), highlighting the contribution of the structural elements (differences in cell shape and texture between the proximal and distal regions) to the visual appearance of the petal. This also confirms that the gene regulatory network regulating pigment production differs from the upstream processes initiating boundary formation during the pre‐patterning phase (Riglet *et al*., 2024).

**Fig. 5:**
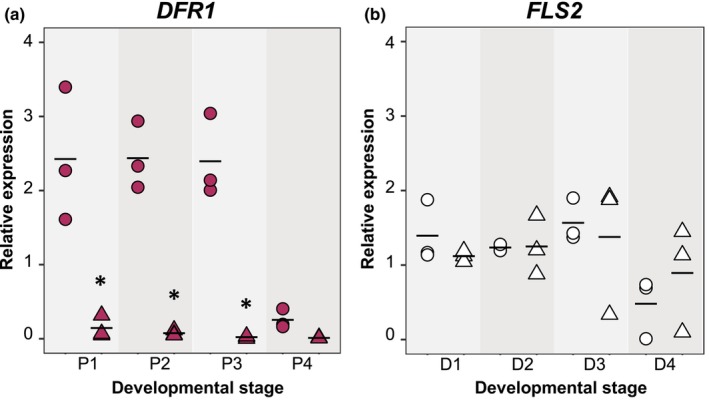
Comparison of expression between *Hibiscus trionum* Cambridge University Botanic Gardens (CUBG) (circles) and *Hibiscus richardsonii* (triangles) of (a) *DFR1* orthologs in proximal petal tissue (P1 to P4) and (b) *FLS2* orthologs in distal petal tissue (D1 to D4). Three biological replicates were extracted per stage. Each data point indicates an average of three technical replicates, and horizontal bars indicate mean relative expression values. Expression values are relative to *HtACTIN1* (*H. trionum* CUBG) or *HrACTIN1* (*H. richardsonii*) expression levels. *, *P* < 0.05 (Student's *t*‐test) of pairwise comparisons made between the same region of *H. trionum* CUBG and *H. richardsonii*.

To identify the genetic changes accounting for reduced anthocyanin production in *H. richardsonii*, we isolated *HrDFR1* and *HrFLS2* in *H. richardsonii*. We did not find any differences in their amino acid sequences compared to the *H. trionum* CUBG orthologs. Next, we measured their expression throughout *H. richardsonii* petal development and found that relative to *H. trionum* CUBG, *HrDFR1* expression was significantly reduced across all petal stages in both proximal (17‐ to 107‐fold lower) and distal (two‐ to ninefold lower) tissues (Figs 5a, S8). The expression profile of *HrFLS2* was similar to *HtFLS2* such that its expression was higher in the distal petal compared to the proximal petal (Figs 5b, S8). Therefore, the reduction of bullseye pigmentation in *H. richardsonii* correlates with a severe reduction in *HrDFR1* expression across the petal primordia.

### Changes at the *BERRY1* locus are responsible for differences in pigmented bullseye size between *H. trionum* CUBG and *H. richardsonii*

MBW complexes, comprising a MYB, a bHLH, and a WD40 component, often regulate the expression of genes encoding key enzymes, such as *DFR* and *FLS* in the flavonoid biosynthesis pathway (Xu *et al*., 2014). We reasoned that some of the TFs regulating *HtDFR1* transcription must be preferentially expressed in the proximal region of the petal. To identify these regulators, we dissected stage 1 petal primordia into proximal and distal regions and performed RNAseq to detect genes differentially expressed just before the emergence of a pigmented bullseye (Fig. S9A; Dataset S1 and S2). Using a fourfold cutoff score, we identified 938 and 343 genes preferentially expressed in the proximal or distal petal regions, respectively (Fig. S9B). Among those *c*. 1300 genes, three MYBs attracted our attention: two were almost exclusively expressed in the proximal petal, which we named *HtBERRY1* and *HtBERRY2*, and one was preferentially expressed in the distal petal that we named *HtCREAM1*. Phylogenetic analysis of the MYB family revealed that the two *HtBERRY* genes belong to subgroup 6 (Fig. S10A; Dataset S3 and S4), a clade that comprises the *A. thaliana PAP1/2* genes and other known regulators of petal pigment production in snapdragon, petunia, monkeyflowers, cotton, *Nigella*, *Clarkia*, and orchids (Schwinn *et al*., 2006; Albert *et al*., 2011; Yuan *et al*., 2014, 2023; Hsu *et al*., 2015; Martins *et al*., 2017; Lin & Rausher, 2021a; Cai *et al*., 2023). *HtCREAM1* fell within a clade comprising cocoa and cotton orthologs but lacked *Arabidopsis* genes (Fig. S10B), making it difficult to predict its role.

**Fig. 6:**
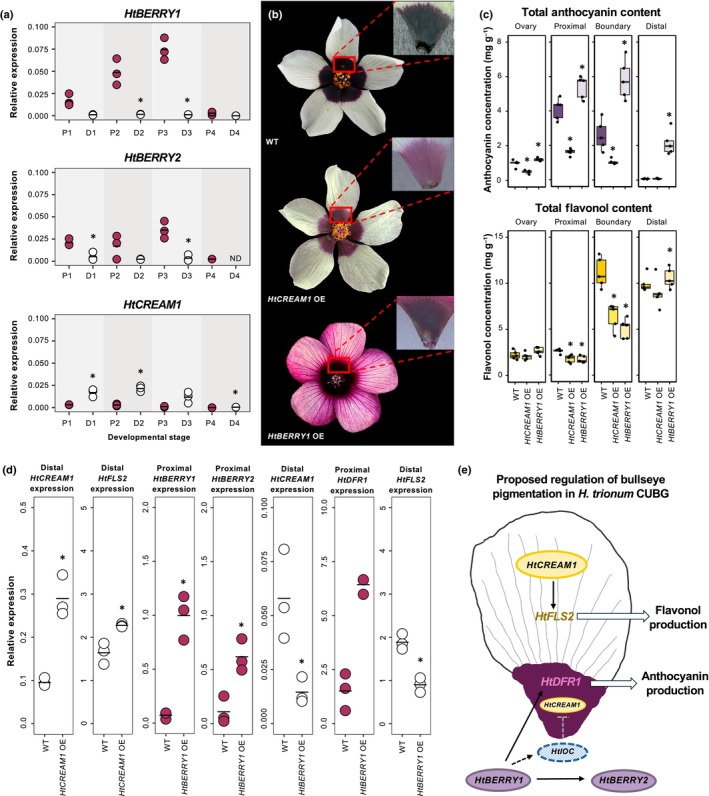
Bullseye pigmentation reflects patterning of *HtBERRY1/2 and HtCREAM1* expression across the petal primordia in *Hibiscus trionum* Cambridge University Botanic Gardens (CUBG). (a) Expression of *HtBERRY1*, *HtBERRY2*, and *HtCREAM1* MYB (myeloblastosis) transcription factors throughout development in proximal (P1 to P4) and distal (D1–D4) petal tissue of *H. trionum* CUBG. ND, non‐detectable expression. *, *P* < 0.05 (Student's or Welch's *t*‐test) pairwise comparisons made between proximal and distal tissue of the same stage. (b) Overexpression (OE) phenotypes of 35S::*HtBERRY1 and* 35S::*HtCREAM1*. Representative images of other independent transgenic lines are provided in Supporting Information Fig. S7. (c) Anthocyanin and flavonol quantification in 35S::*HtBERRY1 and* 35S::*HtCREAM1* lines. Stage 5 flowers were dissected into four regions as highlighted in Fig. 2(a). FIve flowers were analyzed per line. Each data point represents the average of three technical replicates. *, *P* < 0.05 (Student's, Welch's *t*‐test, or Wilcoxon's test) of pairwise comparisons made between *HtCREAM1* OE vs WT and *HtBERRY1* OE vs WT tissue of the same region. (d) Expression of flavonoid‐related structural genes and MYB transcription factors in distal or proximal regions of the overexpression lines. *, *P* < 0.05 (Student's or Welch's *t*‐test) of pairwise comparisons made between the same region against WT and the transgenic line in (c) and (d). (e) Proposed regulation of bullseye pigmentation in *H. trionum* CUBG. A sharp arrow indicates activation, a blunt‐ended arrow indicates repression. Solid lines depict regulatory interactions experimentally tested while dashed lines represent predicted interactions. IOC, Inhibitor of CREAM1. For expression data in (a) and (d), three biological replicates were extracted per stage and each data point indicates an average of three technical replicates, horizontal bars indicate mean relative expression values. Expression values are relative to *HtACTIN1* expression levels.

Quantitative real‐time PCR analysis in *H. trionum* CUBG confirmed the differential expression of *HtBERRY1/2* and *HtCREAM1* along the proximo‐distal axis of the petal throughout development. *HtBERRY1* transcription was always higher in proximal tissue (≥ 15‐fold), increasing until stage 3 before reducing to almost negligible levels (Fig. 6a). *HtBERRY2* expression mirrored that of *HtBERRY1*, but its overall transcription level was lower compared to its paralog. *HtCREAM1* expression complemented *HtBERRY1/2* expression patterns such that its expression was always higher in the distal tissue at early stages (≥ fivefold in the distal region) but dropped off by stage 3 (Fig. 6a).

To investigate the functions of these *MYB* genes and establish their contribution to bullseye pigmentation, we generated *HtBERRY1* and *HtCREAM1* OE lines (Fig. 6b). For both genes, we obtained three independent lines of *35S::HtBERRY1* and *35S::HtCREAM1*. The *35S::HtBERRY1* lines (*HtBERRY1* OE) produced flowers with ectopic pink pigmentation in the distal petal (Figs 6c, S7). Their corollas were entirely colored as the distal region produced 30‐fold more anthocyanin than wild‐type (Fig. 6c); however, a distinct bullseye pattern was still visible (Fig. 6b). This phenotype was consistent with the predicted role of *HBERRY1* as a positive regulator of anthocyanin production. We also measured higher anthocyanin levels in the rest of the petal and ovary (Fig. 6c), further validating HtBERRY1's ability to promote anthocyanin production. Flavonol levels decreased by 30% and 50% in the proximal and boundary regions, respectively, when compared to wild‐type. This could be due to competition between anthocyanin and flavonol production, with the increase in anthocyanin biosynthesis resulting in less precursor available for flavonol synthesis. Flowers from the *35S::HtCREAM1* (*HtCREAM1* OE) lines displayed a paler bullseye (Figs 6b, S7). Flavonoid quantification indicated this was due to reduced anthocyanin production in the proximal region (60% decrease compared to wild‐type; Fig. 6c). Interestingly, the lack of anthocyanin was not accompanied by increased flavonol accumulation. We found that flavonol content was reduced to levels resembling that of the *HtBERRY1* OE line (Fig. 6c) suggesting constitutive *HtCREAM1* OE dampens the entire flavonoid pathway.

To begin dissecting the gene regulatory network controlling pigment production in the *H. trionum* CUBG petal, we measured the expression of the three MYB regulators and the structural genes *HtDFR1* and *HtFLS2* in both *HtBERRY1* and *HtCREAM1* OE lines (Fig. 6d). We found increased *HtFLS2* expression in the distal region of *HtCREAM1* OE petal (1.5‐fold) suggesting HtCREAM1 promotes *HtFLS2* transcription. In the *HtBERRY1* OE line, *HtBERRY1*, *HtBERRY2*, and *HtDFR1* expression in the proximal petal increased by *c*.15‐, six‐, and fourfold compared to wild‐type, respectively. This indicates that HtBERRY1 promotes anthocyanin production through the activation of *HtDFR1* transcription. Interestingly, both *HtCREAM1* and *HtFLS2* expression decreased (fourfold and 1.7‐fold, respectively) in the distal petal when *HtBERRY1* was constitutively overexpressed. HtBERRY1 could repress *HtCREAM1* expression and perhaps *HtFLS2* directly, or more likely indirectly via HtBERRY1 activation of a yet‐to‐be‐identified inhibitor of *HtCREAM1* (IOC) in the distal region. Collectively, our results allow us to propose a small gene regulatory network accounting for the restriction of anthocyanin production to the proximal petal (Fig. 6e).

**Fig. 7:**
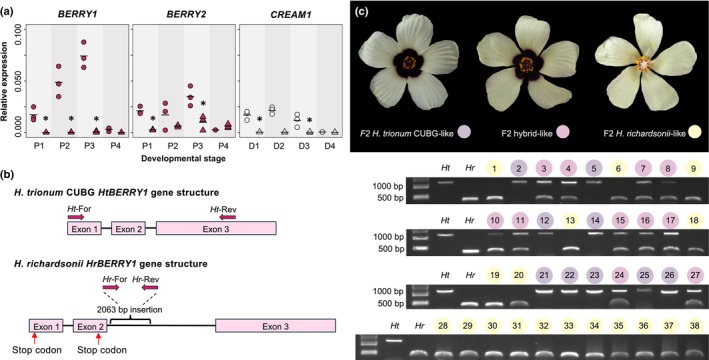
Changes at the *HrBERRY1* locus are responsible for bullseye reduction in *Hibiscus richardsonii*. (a) Comparison of expression between *Hibiscus trionum* Cambridge University Botanic Gardens (CUBG) (circles) and *H. richardsonii* (triangles) of *BERRY1* and *BERRY2* in proximal petal tissue (P1 to P4) and *CREAM1* in distal petal tissue (D1–D4). Each data point indicates an average of three technical replicates, and horizontal bars indicate mean relative expression values. Expression values are relative to *HtACTIN1* (*H. trionum* CUBG) or *HrACTIN1* (*H. richardsonii*) expression levels. *, *P* < 0.05 (Student's *t*‐test) of pairwise comparisons made between the same region of *H. trionum* CUBG and *H. richardsonii*. (b) Sequencing of *HrBERRY1* revealed two premature stop codons in exons one and two and a 2063‐bp insertion in intron two. Allele‐specific primers used in Fig. 7(c) are indicated above each gene structure. (c) Genotyping of the *H. trionum* CUBG (*Ht*) × *H. richardsonii* (*Hr*) F2 population. Blind genotyping of 27 F2 individuals using allele‐specific primers revealed that the nonfunctional *HrBERRY1* allele segregates with the *H. richardsonii‐like* F2 phenotype. An additional 11 F2 individuals with the *H. richardsonii*‐like phenotype were included to further test the segregation hypothesis.

Having identified some of the genetic interactions regulating pigment production in the petal of *H. trionum* CUBG, we hypothesized that one of those interactions or actors of this network was impaired in *H. richardsonii*, interfering with anthocyanin biosynthesis (Fig. 2c). First, the expression of *HrCREAM1* could be enhanced in *H. richardsonii*, repressing anthocyanin production. Alternatively, *HrBERRY* gene expression could be reduced, yielding a smaller pigmented bullseye in *H. richardsonii*. To test these hypotheses, we isolated orthologs of *BERRY1/2* and *CREAM1* in *H. richardsonii* and measured their expression in the developing petals of *H. richardsonii* (Figs 7a, S11). The expression of *HrCREAM1* was 3‐ to 76‐fold lower, not higher, than *HtCREAM1* in both the proximal and distal petals, allowing us to reject the first hypothesis (Figs 7a, S11). *HrBERRY1* expression was scarcely detected, and *HrBERRY2* transcription was reduced by at least 2.5‐fold in the proximal region, likely accounting for the lowered expression of *HrDFR1* in developing petals of *H. richardsonii* (Fig. 5a).

We also examined the coding sequence of each MYB regulator. We identified a single base pair substitution (A>T, 105 bp) in the first exon of *HrCREAM1*, but this was a synonymous mutation encoding a glycine residue at position 35. We found the coding sequence of *HrBERRY1* contained 11 SNPs compared to its ortholog in *H. trionum* CUBG (three substitutions in exon one, four in exon two, and four in exon three) and a large 2063‐bp insertion in the second intron (2445‐bp vs 382‐bp in the second intron of *H. trionum* CUBG). Two of the SNPs within the exons introduced premature stop codons: a T>A substitution at position 17 bp and a G>A substitution at position 159 bp. Thus, the *HrBERRY1* transcript would produce short, truncated proteins that are likely nonfunctional (Fig. 7b). Overall, our findings suggest a scenario where the low expression of *HrDFR1* is caused by a lack of HrBERRY1 activity. If this is the case, the functional *H. trionum* CUBG and nonfunctional *H. richardsonii BERRY1* alleles should segregate with the bullseye size phenotype. To test this hypothesis, we examined allele segregation in the F2 population described in Fig. 3(a). In a blind experiment, 38 F2 individuals were genotyped using allele‐specific primers highlighted in Fig. 7(b). We found that in all 38 F2 individuals, the genotype at the *BERRY1* locus matched the F2 phenotype (Fig. 7c): all *H. trionum* CUBG‐like F2 plants were homozygous for the *HtBERRY1* allele, all *H. richardsonii*‐like F2 individuals were homozygous for the *HrBERRY1* allele, and all hybrid‐like F2 plants carried a copy of each allele. This strongly indicates that the reduced pigmented bullseye of *H. richardsonii* is due to the loss‐of‐function mutations in *BERRY1* or due to a change in a closely linked locus. We examined the genome of *H. trionum* and found that *HtBERRY2* is relatively close to *HtBERRY1* (*c*. 25 kb). However, *BERRY2* is expressed at much lower levels than *BERRY1* in both *H. trionum* and *H. richardsonii* (Fig. S11). Importantly, the two BERRY2 paralogs orthologs are 98% identical (differing in 5 out of 249 amino acids) and *HrBERRY2* appears to code for a fully functional protein, unlike *HrBERRY1*. Hence, we concluded that the reduction in bullseye pigmentation is very likely caused by a nonfunctional *BERRY1* gene in *H. richardsonii*.

### A loss of BERRY1 activity also accounts for the absence of bullseye pigmentation in some populations of *H. verdcourtii*

Based on our phylogenetic reconstruction and distribution of bullseye features across the *Trionum* complex (Fig. 1), the last common ancestor of those four *Hibiscus* species likely exhibited a pigmented bullseye. Reduction in bullseye size then occurred several times during the subsequent evolution of the group, including within *H. verdcourtii*, generating intraspecific variation (Fig. 1b).

**Fig. 8:**
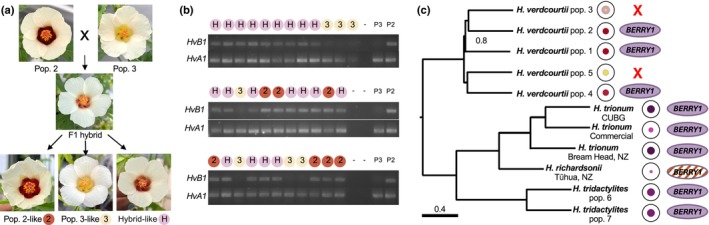
Presence of *HvBERRY1* correlates with pigmented bullseyes in *Hibiscus verdcourtii*. (a) F1 and F2 flower phenotypes of *H. verdcourtii* pop. 2 crossed *with H. verdcourtii* pop. 3. The F1 hybrid produced a flower with a pale pigmented bullseye. The F2 selfing population yielded three flower phenotypes that recapitulated the two parental and hybrid flower morphologies. (b) Genotyping of the *H. verdcourtii* pop. 2 × *H. verdcourtii* pop. 3 F2 population. Blind genotyping of 34 F2 individuals using *HvBERRY1*‐specific primers revealed that the presence of *HvBERRY1* segregates with a pigmented bullseye. Letters and numbers above gel lanes provide the flower phenotype corresponding to each genotyped F2 individual: H, hybrid‐like flower; 2, *H. verdcourtii* pop. 2‐like flower; and 3, *H. verdcourtii* pop. 3‐like flower. The parental lines (P2, *H. verdcourtii* pop. 2 and P3, *H. verdcourtii* pop. 3) and no‐template control (−) were included as controls. PCR results using different primer pairs are given in Supporting Information Fig. S12 (c). Multiple independent losses of the pigmented bullseye in the *Hibiscus trionum* complex are a result of modifications to the *BERRY1* locus. In the *H. verdcourtii* clade, loss of *HvBERRY1* is correlated with a flower phenotype lacking a pigmented bullseye. In *H. richardsonii*, *HrBERRY1* transcripts are detected at very low levels, and several mutations within the coding region likely render the corresponding protein nonfunctional. Numbers next to nodes indicate support values. Branch lengths are shown in coalescent units.

Interestingly, we amplified *HvBERRY1* in populations 1, 2, and 4 yielding individuals with red bullseye flowers, but we failed to amplify this gene in populations 3 and 5 whose flowers are devoid of red pigmentation at the petal base (Fig. 1b). To determine whether *BERRY1* was involved in *H. verdcourtii* pigmentation variation, we crossed red bullseye individuals from population 2 with plants from population 3 lacking a pigmented bullseye (Fig. 8a). The F1 hybrid produced flowers resembling those from population 2 (Fig. 8a), indicating that the pigmented bullseye trait is also dominant in *H. verdcourtii*. We found that bullseye color segregated in a 3 : 1 ratio in the F2 population as 37 and 11 individuals displayed a red and pale bullseye phenotype, respectively, leading us to conclude that pigmentation loss in *H. verdcourtii* was due to a change affecting a single locus. To test whether *HvBERRY1* could be the causative locus, we performed blind genotyping of 34 F2 individuals (Fig. 8b). Our results demonstrate that the presence/absence of *HvBERRY1* is a perfect predictor of the presence/absence of a red bullseye (Fig. 8b), supporting the idea that the loss of *HvBERRY1* accounts for the loss of petal pigmentation in *H. verdcourtii* (Figs 8c, S12).

### Preference assays reveal bumblebees favor larger pigmented bullseyes

The fact that sister species and distinct populations from the same species exhibit pigmented bullseyes of contrasting sizes raises the interesting possibility that a change in pattern dimension could influence pollinator selection, reduce genetic exchange between populations and thus play a role in reproductive isolation and speciation. We previously showed that buff‐tailed bumblebees (*B. terrestris* v. audax) can discriminate between 3D‐printed plastic disks mimicking the proportions of *H. trionum* CUBG and *H. richardsonii* bullseyes and have an innate preference for disks with the larger bullseye (Riglet *et al*., 2024). Real flowers, however, are complex structures with differences in shape, scent, and temperature that, along with pigmentation variation, may also influence pollinator responses. To test bumblebee behavior in a more realistic setting, we conducted preference test experiments using real flowers from each species. We found that *B. terrestris* chose to land on *H. trionum* CUBG flowers over *H. richardsonii* 85% of the time (*n* = 40; 34 bees selected *H. trionum* CUBG vs six bees selected *H. richardsonii*, *P* = 3.51 × 10^−7^, one‐sample *t*‐test), indicating that the preference for the larger bullseye stands in a real‐flower context.

## Discussion

### Resolving the phylogenetic history of the *Trionum* complex sheds light on bullseye pattern evolution

Relationships within the *Hibiscus* genus are historically difficult to resolve (Pfeil & Crisp, 2005; Hanes *et al*., 2024). Here, we demonstrate how phylogenomic approaches can help illuminate the evolutionary history of previously recalcitrant groups like the *Trionum* complex. Our phylogenetic reconstruction divided the *Trionum* complex into four distinct clades and positioned *H. richardsonii* as a sister species to *H. trionum*. This finding corroborates previous morphological and ecological studies where the status of *H. richardsonii* as a separate species or a variant of *H. trionum* was debated (de Lange, 2008; Craven *et al*., 2011).

Resolving the relationships within the *Trionum* complex also enabled us to illuminate the evolution of petal patterning. We found that cell shape distribution was fixed across the complex, as conical cells were exclusively located in the distal region while tabular cells were only present in the proximal petal. Unlike the other members of the complex, *H. verdcourtii* lacked striations in the proximal petal region regardless of the presence of a pigmented bullseye (Fig. S2). This indicates the genetic mechanisms controlling cell shape, cuticle texture, and pigmentation are distinct and the ability to produce a striated cuticle was gained after the divergence of *H. verdcourtii* or lost along the lineage leading to *H. verdcourtii*. Our analysis established pigmentation as the most labile feature of epidermal petal cells, as accessions with a reduced pigmented bullseye did not cluster together in the species tree. Such versatility in petal pigmentation suggests it is an important feature undergoing selection and contributes to bullseye evolution.

### *HtBERRY1* plays an important role in the production of a pigmented bullseye in *Hibiscus*

In *Dianthus caryophyllus*, *Rosa multiflora*, and *Camellia japonica* flowers, expression of *DFR* and *FLS* homologs correlates with red and white flowers, respectively (Luo *et al*., 2016). In *Clarkia gracilis*, *DFR* expression patterns coincide with petal spot formation during development (Martins *et al*., 2013). Here, we also demonstrate compartment‐specific expression of *DFR* and *FLS* before the emergence of pigmentation patterns at very early stages of development (stage 1). Our results are consistent with recently published transcriptome data for later stages of petal development (stages 3–4) in *H. trionum* (Koshimizu *et al*., 2023). Our findings indicate strong preferential expression of *HtDFR1* in the proximal petal region is central to restricting anthocyanin production to the petal base. Other flavonoid pathway genes (chalcone synthases, flavonoid 3′‐hydroxylases, flavonoid 3′,5′‐hydroxylases, and ANSs) are either uniformly present across the petal or slightly higher in the distal domain (Fig. S13). Our results also argue for functional divergence between the three *DFR* genes and between the three *FLS* paralogs. Functional divergence due to changes in enzymatic capabilities was reported for *FLS* paralogs in rapeseed (*Brassica napu*s) and *Arabidopsis* (Owens *et al*., 2008; Schilbert *et al*., 2021) and for *DFR* homologs in morning glory (*Ipomoea* sp.; Des Marais & Rausher, 2008). In *Hibiscus trionum*, such diversification involved divergence of expression patterns between paralogs, but whether this was also accompanied by changes in coding sequences affecting the biochemical properties of the enzymes they code for remains to be elucidated.

The restriction of *HtDFR1* activity to the petal base is due in part to the localized expression of one of its activators, *HtBERRY1*, to the proximal petal region, as constitutive overexpression of *HtBERRY1* is sufficient to trigger ectopic expression of *HtDFR1* and yield anthocyanin production in the distal region. We found that the close paralog of *HtBERRY1*, *HtBERRY2*, is also preferentially expressed in the proximal petal region, although at much lower levels than *HtBERRY1*. Both corresponding proteins are highly similar (82.9% identity, 94.8% similarity) suggesting that HtBERRY2 could also participate in *HtDFR1* regulation and pigment production. It will be interesting to test this hypothesis in the future and determine whether the residual expression of *HrDFR1* in the absence of a functional HrBERRY1 is due to the activity of HrBERRY2. Interestingly, the coloration obtained in the distal domain in the *HtBERRY1* OE line was not as intense as in the proximal domain, and anthocyanin levels remained lower (Fig. 4d–f). This could be due to several factors. First, the lack of or restricted availability of a HtBERRY1 partner could limit its ability to activate *HtDFR1* expression in the distal region. Alternatively, other TFs contributing to *HtDFR1* expression could be absent in the distal region. Secondly, differences in the chromatin landscape at the *HtDFR1* promoter could impair the effective binding of HtBERRY1 to *cis*‐regulatory elements. However, our expression data (Figs 6d, S14) suggest that *HtDFR1* is expressed at similar levels in both proximal and distal regions in the *HtBERRY1* OE line and rule out both hypotheses. Instead, limited expression of enzymes acting downstream of *HtDFR1* in the same region could account for dampened anthocyanin production. We found that apart from *ANS*, other anthocyanin biosynthesis genes were uniformly present throughout the developing petal primordia (Fig. S13). *HtANSa* is expressed at high levels in both petal domains but slightly higher in the proximal domain (less than twofold difference). Therefore, it is possible that increasing *HtANSa* expression in the distal petal domain could boost color intensity, but it is unlikely a limiting factor for anthocyanin production when *HtBERRY1* is overexpressed. Finally, competition with *HtFLS2* for precursor availability could prevent *HtDFR1* from synthesizing anthocyanin effectively in the distal petal region. The fact that *HtCREAM1* and *HtFLS2* are both downregulated in the distal region of *HtBERRY1* OE makes it unlikely that competition between *HtDFR1* and *HtFLS2* is the main cause of the paler hue of the distal domain of the *HtBERRY1* OE line. Taken together, our results suggest that HtBERRY1 promotes pigment production by at least two different routes: first, by inducing the expression of *HtDFR1*, and second, by reducing the transcription of *HtCREAM1* and *HtFLS2*, limiting possible competition between *HtDFR1* and *HtFLS2* in the proximal region and thus favoring anthocyanin synthesis over flavonol production in the center of the bullseye.

Whether HtBERRY1 directly represses *HtCREAM1* and/or *HtFLS2* transcription remains to be tested. If this is the case, HtBERRY1 must be able to act as both an activator and a repressor by forming different MBW complexes involving different bHLH partners. The R2R3 MYB subfamily, to which HtBERRY1 belongs, is the largest of the four MYB families and can be characterized by two adjacent imperfect MYB repeats (Wu *et al*., 2022). Aside from other functions, over half of R2R3 MYBs are involved in the regulation of specialized metabolism such as phenylpropanoid biosynthesis, which results in end products such as flavonoids. The importance of MYB regulation in the flavonoid pathway has been highlighted across several species, including *Petunia hybrida* and *Antirrhinum majus*. PhDPL, PhPHZ, AmROSEA1/2, and AmVENOSA all induce the expression of *DFR* orthologs (Schwinn *et al*., 2006; Albert *et al*., 2011; Shang *et al*., 2011). However, a dual role for a single R2R3 MYB as a transcriptional activator and repressor has not been previously reported. Alternatively, HtBERRY1 could act indirectly by activating the expression of a repressor that downregulates *HtCREAM1* and *HtFLS2*. Overall, our results highlight that HtBERRY1 plays an important role in controlling anthocyanin production across the petal surface during bullseye formation.

Studies in a range of species have shown that the spatial control of *MYB* genes is central to the emergence of pigmentation patterns in many angiosperms (Ding, 2023). Unfortunately, little is known regarding the upstream regulators of R2R3 MYBs during flower development. In snapdragon, anthocyanin production is controlled by the R2R3 MYB VENOSA and its bHLH partner DELILA. *DELILA* is expressed throughout the petal epidermis, while *VENOSA* is only transcribed around the vasculature such that pigment production is limited to the epidermal cells directly above the vasculature where both VENOSA and DELILA are present (Shang *et al*., 2011). A signal emerging from differentiating vascular cells could induce local *VENOSA* expression, or the same upstream signal that specifies vasculature formation (e.g. auxin signaling) could in parallel promote *VENOSA* transcription. In the *Trionum* complex, the bullseye pattern exhibits strong polarity along the petal proximo‐distal axis, reflecting the differential expression of *HtBERRY1*. Hence, cues that specify and pattern the proximo‐distal axis of lateral organs are likely to act as upstream regulators of *HtBERRY1* expression. Interestingly, the venation patterning in snapdragon is prominent in the distal corolla but largely lacking in the proximal region (Shang *et al*., 2011). This suggests that the ability of an epidermal cell to activate anthocyanin production is dependent on its position both relative to the vasculature and along the petal length. Therefore, these positional signals are not mutually exclusive and can be combined to enable cells to perceive and integrate spatial information from multiple inputs. The processes that drive proximo‐distal polarity establishment in petal primordia remain elusive (Salvi & Moyroud, 2025), but future studies aiming at uncovering the mechanisms that spatially restrict *HtBERRY1* transcription during petal development represent a promising avenue to shed light on these obscure processes.

### Independent modifications of a single locus lead to repeated reductions in bullseye dimensions

The occurrence of forms closely resembling each other across the tree of life supports the notion that similar features repeatedly arose independently through evolution. However, the molecular basis supporting phenotypical convergence is often unclear. Here, we used a reduction in petal pattern dimensions across a small group of *Hibiscus*, the *Trionum* complex, to unravel the molecular underpinnings of reduction in bullseye pigmentation. Our results uncovered a case of replicated evolution, where the size of the petal‐pigmented area shrunk several times independently across the *Trionum* complex. In at least three cases, this bullseye reduction was due to distinct changes affecting the same MYB‐encoding locus, *BERRY1*, triggering either a reduction in expression and premature stop codons of *HrBERRY1* in *H. richardsonii* or complete gene loss of *HvBERRY1* from the genome of two populations of *H. verdcourtii*. Occurrences of replicated evolution driving the pigmentation changes have been documented in both plant and animal species. Geographically isolated populations of agouti‐colored beach mice share an independently selected allele contributing to the cryptic coloration that assists in protection against predators (Wooldridge *et al*., 2022). The transition to red flower color in *Ipomea* and the shift from blue to red flowers of Andean *Iochroma* have been observed multiple times (Streisfeld & Rauser, 2009; Smith & Rausher, 2011). In both cases, the change in flower color was accompanied by mutations affecting flavonoid hydroxylases that redirect the anthocyanin biosynthesis pathway toward the production of pelargonidin derivatives (red pigments).

Findings by Wheeler *et al*., 2022 suggest regulators of the flavonoid pathway evolve faster than structural genes. In this study, we demonstrate clear instances of replicated evolution that impact petal patterning through independent modifications to the BERRY1 TF, rather than through changes affecting *DFR* or *FLS* loci. To date, all studies examining floral pigment pattern production highlight that restriction of MBW complex activity to specific petal regions is central to the production of a pigmented motif on the petal surface, and this is often achieved by precisely controlling *MYB* expression (Albert *et al*., 2014). Consistently, changes in petal patterning during evolution often rely on the genetic variation of MYB TFs. In *M. lewisii*, a two‐component activator–inhibitor system involving two MYB TFs regulates pigmentation spots on *Mimulus* corollas. Genetic variation in *RTO* encoding the MYB repressor component accounts for the presence of both spotted and red tongue petal pattern phenotypes in natural populations (Ding *et al*., 2020). R2R3 MYBs, rather than their bHLH partners, are the main drivers of localized anthocyanin synthesis in *Antirrhinum*, petunia, and maize (Piazza *et al*., 2002; Schwinn *et al*., 2006; Albert *et al*., 2011). Furthermore, variation affecting the expression or functionality of subgroup 6 MYBs in *Clarkia* is sufficient to explain petal pattern variation across the genus (Lin & Rausher, 2021a,b). Why MYBs rather than bHLHs tend to limit the production of pigment to subregions of the epidermis is unclear. It could reflect fundamental differences in the transcriptional regulation of both families or instead indicate that the MYB components are less likely to have pleiotropic effects than their bHLH partner. Together, these findings underscore that evolution often recycles molecular mechanisms to generate petal diversity, revealing that the investigation of regulatory networks provides critical insights into how repeated evolutionary outcomes are achieved.

Our findings also suggest that changes in pattern dimensions may have contributed to the diversification of the *Trionum* complex by impacting plant–pollinator interactions as bumblebees exhibit a strong innate preference for the larger bullseye of *H. trionum* CUBG over the reduced one of its sister species *H. richardsonii*. The geographical distributions of *H. trionum* and *H. richardsonii* overlap in the North Island of New Zealand (Craven *et al*., 2011), and we have shown the two species are cross‐fertile when hand‐pollinated in greenhouse conditions (Fig. 3a). In the future, it will be important to determine whether hybridization mediated by pollinating insects also occurs in the wild or whether the difference in bullseye size, combined with other factors, favors reproductive isolation.

Studies in silverweed and sunflowers suggest that floral bullseye patterns can also provide protection against UV damage and desiccation (Koski & Ashman, 2015; Todesco *et al*., 2022). Whether the evolutionary history of the *Hibiscus* bullseye was shaped by environmental factors is not yet known, but adaptations to abiotic cues could further explain the variation in pigmented bullseyes in natural populations. Future investigations within the natural habitat of these *Hibiscus* species will provide valuable information such as the identification of species‐specific pollinators, if any, and the frequency of self‐fertilization events associated with possible low pollinator availability.

## Competing interests

None declared.

## Author contributions

EM conceptualized and designed the project. MTSY, ALMF, VT, SZ and LR performed experiments and data analysis. MTSY validated experiments and performed statistical analysis. JFW analyzed the transcriptome data and performed the phylogenomic analysis. MTSY and EM prepared figures and wrote the manuscript, with input from all authors.

## Disclaimer

The New Phytologist Foundation remains neutral with regard to jurisdictional claims in maps and in any institutional affiliations.

## Supporting information


**Dataset S1** Assembled transcripts from *Hibiscus trionum*, stage 1 petals.

**Dataset S2** Differential gene expression analysis between proximal and distal regions of stage 1 *Hibiscus trionum* petal primordia.

**Dataset S3** Amino acid sequences of the 483 MYB sequences used to assess the phylogenetic placement of HtBERRY1, HtBERRY2, and HtCREAM1.

**Dataset S4** Complete MYB family tree generated using the 483 sequences from Dataset S3.

**Fig. S1** Geographical location of *Hibiscus* species/populations used in this study.
**Fig. S2** Cell shape and texture across the adaxial petal epidermis of the 11 different accessions from the *Trionum* complex used in this study.
**Fig. S3** Comparison of pigmentation throughout petal development in *Hibiscus trionum* CUBG and *Hibiscus richardsonii*.
**Fig. S4** Light microscopy images of *Hibiscus trionum* CUBG and *Hibiscus richardsonii* boundary cell types.
**Fig. S5** Expression of *HtDFR2*, *HtDFR3*, *HtFLS1 and HtFLS2* throughout flower development in proximal and distal petal tissue of *Hibiscus trionum* CUBG.
**Fig. S6** Normalized read counts from RNAseq data for *HtDFR* homologs and *HtFLS* homologs in proximal and distal petal tissue of *Hibiscus trionum* CUBG at stage 1 and stage 2.
**Fig. S7** Transgene expression level in various *HtDFR1*, *HtBERRY1* and *HtCREAM1 Hibiscus trionum* CUBG lines.
**Fig. S8** Expression of *HrDFR1* and *HrFLS2* throughout flower development in proximal and distal petal tissue of *Hibiscus richardsonii*.
**Fig. S9** Identification of differentially expressed genes between the proximal and distal regions of stage 1 petal primordia in *Hibiscus trionum* CUBG.
**Fig. S10** Placement of *HtBERRY1*, *HtBERRY2* and *HtCREAM1* within the MYB family phylogenetic tree.
**Fig. S11** Expression of *HrBERRY1*, *HrBERRY2* and *HrCREAM1* throughout development in proximal and distal petal tissue of *Hibiscus richardsonii*.
**Fig. S12** Predicted gene structure of *HvBERRY1* in *Hibiscus verdcourtii* with additional allele‐specific primers.
**Fig. S13** Normalized read counts for flavonoid‐related structural genes, subgroup IIIf bHLH and *TTG1/LWD40* homologs in *Hibiscus trionum* CUBG Stage 1 and 2 petal tissue.
**Fig. S14** Expression of flavonoid‐related structural and MYB transcription factors in distal or proximal regions of wild‐type and *HtBERRY1* OE or *HtCREAM1* OE *Hibiscus trionum* CUBG lines.
**Table S1** *Hibiscus* species used in this study.
**Table S2** *Hibiscus trionum* CUBG transgenic lines generated in this study.
**Table S3** Primer sequences used in this study.
**Table S4** Plant expression vectors generated for this study.
Please note: Wiley is not responsible for the content or functionality of any Supporting Information supplied by the authors. Any queries (other than missing material) should be directed to the *New Phytologist* Central Office.

## Data Availability

The coding and genomic sequences of all *H. trionum* genes mentioned in this study can be accessed via the genome of *H. trionum* available on GenBank (https://www.ncbi.nlm.nih.gov/datasets/genome/GCA_030270665.1/) using the HRI_ reference numbers provided in the Material and Methods sections. The coding sequences of *HrBERRY1*, *HrDFR1*, *HrFLS2*, and *HvBERRY1* have been deposited in GenBank under the accession nos. PQ456077, PQ450317, PQ450318, and PQ450319, respectively. Transcriptomic data associated with the phylogenomic analysis of the Trionum complex (accession nos. SAMN47801648 to SAMN47801669) and the comparative analysis of gene expression between proximal and distal petal regions of H. trionum stage 1 petal (accession nos. SAMN47793371 to SAMN47793390) have been deposited on GenBank SRA (https://www.ncbi.nlm.nih.gov/sra). The amino acid sequences used for assessing the placement of HtBERRY1, HtBERRY2, and HtCREAM1 in the MYB family phylogeny are provided in Dataset S3. The algorithms and codes used for phylogenomic analyses, RNA‐seq data analysis, and differential gene expression analysis have been published elsewhere, as indicated in the references provided in the Material and Methods section.
