# Supplementary material for: The genetic basis of replicated bullseye pattern reduction across the Hibiscus trionum complex
Source: New Phytol. 2025 May 20;247(2):863–83. doi: 10.1111/nph.70168 (PMC12177274; doi:10.1111/nph.70168)
Supplement: Supplementary file 5 — Fig. S1 Geographical location of Hibiscus species/populations used in this study. Fig. S2 Cell shape and texture across the adaxial petal epidermis of the 11 different accessions from the Trionum complex used in this study. Fig. S3 Comparison of pigmentation throughout petal development in Hibiscus trionum CUBG and Hibiscus richardsonii. Fig. S4 Light microscopy images of Hibiscus trionum CUBG and Hibiscus richardsonii boundary cell types. Fig. S5 Expression of HtDFR2, HtDFR3, HtFLS1 and HtFLS2 throughout flower development in proximal and distal petal tissue of Hibiscus trionum CUBG. Fig. S6 Normalized read counts from RNAseq data for HtDFR homologs and HtFLS homologs in proximal and distal petal tissue of Hibiscus trionum CUBG at stage 1 and stage 2. Fig. S7 Transgene expression level in various HtDFR1, HtBERRY1 and HtCREAM1 Hibiscus trionum CUBG lines. Fig. S8 Expression of HrDFR1 and HrFLS2 throughout flower development in proximal and distal petal tissue of Hibiscus richardsonii. Fig. S9 Identification of differentially expressed genes between the proximal and distal regions of stage 1 petal primordia in Hibiscus trionum CUBG. Fig. S10 Placement of HtBERRY1, HtBERRY2 and HtCREAM1 within the MYB family phylogenetic tree. Fig. S11 Expression of HrBERRY1, HrBERRY2 and HrCREAM1 throughout development in proximal and distal petal tissue of Hibiscus richardsonii. Fig. S12 Predicted gene structure of HvBERRY1 in Hibiscus verdcourtii with additional allele‐specific primers. Fig. S13 Normalized read counts for flavonoid‐related structural genes, subgroup IIIf bHLH and TTG1/LWD40 homologs in Hibiscus trionum CUBG Stage 1 and 2 petal tissue. Fig. S14 Expression of flavonoid‐related structural and MYB transcription factors in distal or proximal regions of wild‐type and HtBERRY1 OE or HtCREAM1 OE Hibiscus trionum CUBG lines. Table S1 Hibiscus species used in this study. Table S2 Hibiscus trionum CUBG transgenic lines generated in this study. Table S3 Primer sequences used i [file NPH-247-863-s001.docx]

***New Phytologist* Supporting Information**

Article title: The genetic basis of replicated bullseye pattern reduction across the Trionum Complex

Authors: May T. S. Yeo^1,2^, Alice L. M. Fairnie^1,3^, Valentina Travaglia^1,4^, Joseph F. Walker^1,5^, Lucie Riglet^1^, Selin Zeyrek^1,2^, and Edwige Moyroud^1,2, *^

Article acceptance date: 2 April 2025

This following Supporting Information is available for this article:

**Fig. S1** Geographical location of *Hibiscus* species/populations used in this study.

**Fig. S2** Cell shape and texture across the adaxial petal epidermis of the 11 different accessions from the Trionum Complex used in this study.

**Fig. S3** Comparison of pigmentation throughout petal development in (A) *H. trionum* CUBG and (B) *H. richardsonii*.

**Fig. S4** Light microscopy images of *H. trionum* CUBG and *H. richardsonii* boundary cell types

**Fig. S5** Expression of *HtDFR2, HtDFR3, HtFLS1 and HtFLS2* throughout flower development in proximal and distal petal tissue of *H. trionum* CUBG.

**Fig. S6** Normalized read counts from RNAseq data for *HtDFR* homologs and *HtFLS* homologs in proximal and distal petal tissue of *H. trionum* CUBG at stage 1 and stage 2.

**Fig. S7** Transgene expression level in various *HtDFR1, HtBERRY1* and *HtCREAM1* *H. trionum* CUBG lines.

**Fig. S8** Expression of *HrDFR1* and *HrFLS2* throughout flower development in proximal and distal petal tissue of *H. richardsonii*.

**Fig. S9** Identification of differentially expressed genes between the proximal and distal regions of stage 1 petal primordia in *H. trionum* CUBG.

**Fig. S10** Placement of *HtBERRY1*, *HtBERRY2* and *HtCREAM1* within the MYB family phylogenetic tree.

**Fig. S11** Expression of *HrBERRY1, HrBERRY2* and *HrCREAM1* throughout development in proximal and distal petal tissue of *H. richardsonii*.

**Fig. S12** Predicted gene structure of *HvBERRY1* in *H. verdcourtii* with additional allele-specific primers.

**Fig. S13** Normalized read counts for flavonoid-related structural genes, subgroup IIIf bHLH and *TTG1/LWD40* homologs in *H. trionum* CUBG Satge 1 and 2 petal tissue.

**Fig. S14** Expression of flavonoid-related structural and MYB transcription factors in distal or proximal regions of wildtype and *HtBERRY1* OE or *HtCREAM1* OE *H. trionum* CUBG lines.

**Table S1** *Hibiscus* species used in this study.

**Table S2** *H. trionum* transgenic lines generated in this study.

**Table S3** Primer sequences used in this study.

**Table S4** Plant expression vectors generated for this study.

The following four Supplementary Data files are provided as separate files:

**Supplementary Data 1**. Assembled transcripts from *H. trionum*, stage 1 petals. Data related to Fig S9.

**Supplementary Data 2**. Differential gene expression analysis between proximal and distal regions of stage 1 *H. trionum* petal primordia. Data related to Fig S9.

**Supplementary Data 3**. Amino acid sequences of the 483 MYB sequences used to assess the phylogenetic placement of HtBERRY1, HtBERRY2 and HtCREAM1. Data related to Fig S10.

**Supplementary Data 4**. Complete MYB family tree generated using the 483 sequences from Supplementary Data 3. Data related to Fig S10.

**Fig. S1.** Geographical location of *Hibiscus* species/populations used in this study. Wildtype *H. trionum* and *H. richardsonii* were isolated from Bream Head and Mayor Island (Tūhua), New Zealand, respectively. *H. verdcourtii* and *H. tridactylites* populations were isolated from various sites in Queensland and New South Wales, Australia (Craven et al., 2011).

**Fig. S2.** Cell shape and texture across the adaxial petal epidermis of the 11 different accessions from the Trionum Complex used in this study (see Fig. 1 and Fig. S1). Unless stated, all nodes are perfectly supported. Branch lengths are shown in coalescent units. All accessions display tabular cells in the proximal petal region and smooth conical cells in the distal region. The tabular cells in the proximal region are covered with a striated cuticle, except for the five *H. verdcourtii* accessions that display smooth tabular cells in the proximal region. Scale bar = 50uM.

**Fig. S3.** Comparison of pigmentation throughout petal development in (A) *H. trionum* CUBG and (B) *H. richardsonii*. Images show adaxial epidermis of petal primordia from Stage 1 (S1) to Stage 5 (S5) as described in Moyroud *et al.,* 2022. Scale bar = 1mm in S1-S4 and 5mm in S5.

**Fig. S4.** Light microscopy images of *H. trionum* CUBG and *H. richardsonii* boundary cell types (middle and bottom rows). Cartoon symbols (top row) are used to describe F1 and F2 boundary phenotypes in Fig. 3. Scale bars = 50uM.

**Fig. S5.** Expression of *HtDFR2, HtDFR3, HtFLS1 and HtFLS2* throughout flower development in proximal (P1 to P4, purple dots) and distal (D1 to D4, white dots) petal tissue of *H. trionum* CUBG. Three biological replicates were extracted per stage and each data point indicates an average of three technical replicates, horizontal bars indicate mean relative expression values. Expression values are relative to *HtACTIN1* expression levels.

**Fig. S6.** Normalized read counts from RNAseq data for *HtDFR* homologs and *HtFLS* homologs in proximal (P1 and P2) and distal (D1 and D2) petal tissue of *H. trionum* CUBG at stage 1 and stage 2 (before and after bullseye pigmentation emergence, respectively). The y-axis shows the normalized read counts in Transcripts Per Million (TPM). Error bars = +/- SD with n = 5 independent biological replicates.

**Fig. S7.** Transgene expression level in various *HtDFR1, HtBERRY1* and *HtCREAM1* *H. trionum* CUBG lines. In *HtBERRY1* OE line #2, severe defects to floral development were observed (reduced growth of all floral organs and sterility of reproductive organs). Floral development was normal in other lines and sterility appeared to correlate with the strength of ectopic pigmentation suggesting constitutive overexpression of *HtBERRY1* and/or abnormal production of anthocyanin levels were associated with sterility. Each column indicates an average of three biological replicates, error bars represent +/- SD. Expression values are relative to *HtACTIN1* expression levels. WT = *H. trionum* CUBG.

**Fig. S8.** Expression of *HrDFR1* and *HrFLS2* throughout flower development in proximal (P1 to P4) and distal (D1 to D4) petal tissue of *H. richardsonii*. Three biological replicates were extracted per stage. Each data point indicates an average of three technical replicates, horizontal bars indicate mean relative expression values. Expression values are relative *HrACTIN1* expression levels.

**Fig. S9.** Identification of differentially expressed genes between the proximal and distal regions of stage 1 petal primordia in *H. trionum* CUBG. (**A**) Image of a dissected *H. trionum* CUBG stage 1 petal showing the two regions used for RNA extraction and transcriptome analysis: region above the red dotted line = distal region, region below the red dotted line = proximal region. (**B**) Volcano plot displaying genes identified in the transcriptome analysis of stage 1 petals. Genes preferentially expressed in the proximal region (>4x difference compared to distal region) are represented by purple dots while genes preferentially expressed in the distal region (>4x difference compared to proximal region) are represented by white dots. The blue lines represent the log2-fold threshold used for differential expression cut-off. Genes considered not differentially expressed between proximal and distal regions are depicted with black dots. Data associated with complete differential gene expression analysis and transcript assembly are provided as Supplementary Data 1 and the complete phylogeny is provided as Supplementary Data 2.

**Fig. S10.** Placement of *HtBERRY1*, *HtBERRY2* and *HtCREAM1* within the MYB family phylogenetic tree. (**A**) Phylogenetic tree depicting the position of *HtBERRY1 and HtBERRY2* within the subgroup 6 R2R3-MYB cluster. (**B**) Phylogenetic tree depicting homology relationships between *HtCREAM1* and its three *H. trionum* paralogs and R2R3-MYBs from cotton and cocoa. *H. trionum* CUBG genes are highlighted in purple, cotton (*Gossypium raimondii*) genes are highlighted in blue, cocoa (*Theobroma cacao*) genes are highlighted in brown and *Arabidopsis thaliana* genes are boxed in green. Ultrafast Bootstraps values are given next to each node. Scale bar represents number of changes per site. Subgroup 6 R2R3-MYBs from other species, known to regulate anthocyanin production in the flowers of other species have also been included in (A). *Antirrhinum majus*: ROSEA1, ROSEA2, VENOSA; *Mimulus lewisii*: PELAN, NEGAN; *Mimulus guttatus*: MYB113-like; *Petunia hybrida*: DEEP PURPLE, PURPLE HAZE. The trees presented in A and B are part of a larger R2R3-MYB phylogenetic analysis we conducted, including all known R2R3-MYBs from *A. thaliana*, *G. raimondii* and *T. cacao*. The sequences used for this analysis are provided a Supplementary Data 3 and the complete phylogeny is provided as Supplementary Data 4.

**Fig. S11.** Expression of *HrBERRY1, HrBERRY2* and *HrCREAM1* throughout development in proximal (P1 to P4) and distal (D1 to D4) petal tissue of *H. richardsonii*. Three biological replicates were extracted per stage and each data point indicates an average of three technical replicates, horizontal bars indicate mean relative expression values. Expression values are relative to *HrACTIN1* expression levels.

**Fig. S12.** Predicted gene structure of *HvBERRY1* in *H. verdcourtii* with additional allele-specific primers. *HvBERRY1* can be amplified from *H. verdcourtii* pops. 1, 2 and 4 but not from pop 3 or 5. Hv-F2 and Hv-R1 primers were used to genotype the F2 population as described in Fig. 8B. (-) = no template control.

**Fig. S13.** Normalized read counts for flavonoid-related structural genes, subgroup IIIf bHLH and *TTG1/LWD40* homologs in *H. trionum* CUBG Stage 1 and 2 petal tissue. **(A)** *CHS* orthologs, **(B)** *HtF3’H, HtF3’5Ha, HtF3’5Hb, HtANSa, HtANSb* **(C)** subgroup IIIf *bHLH* and **(D)** *TTG1/LWD40* genes expressed in proximal (P1 and P2) and distal (D1 and D2) petal tissue of *H. trionum* CUBG at stage 1 and stage 2 (before and after bullseye pigmentation emergence, respectively). The y-axis shows the normalized read counts in Transcripts Per Million (TPM). Error bars = +/- SD with n = 5 independent biological replicates.

**Fig. S14.** Expression of flavonoid-related structural and MYB transcription factors in distal (white dots) or proximal (purple dots) regions of wildtype and (A) *HtBERRY1* OE or (B) *HtCREAM1* OE *H. trionum* CUBG lines. Three biological replicates were extracted per stage and each data point indicates an average of three technical replicates, horizontal bars indicate mean relative expression values. Expression values are relative to *HtACTIN1* expression levels.

| **Species** | **Description** | **Provenance** | **Voucher** |
| --- | --- | --- | --- |
| *H. trionum* L. | Wildtype | Cambridge University Botanic Garden collection | CGE00046422 |
| *H. trionum* L. | Diploid New Zealand naturalised race | Bream Head, New Zealand | AK253689, CGE00046417 |
| *H. trionum* L. | Commercial | Netherlands | CGE00080883 |
| *H. richardsonii* Sweet ex Lindl. | Wildtype | Mayor Island (Tūhua), New Zealand | AK251841, CGE00046420 |
| *H. verdcourtii* Craven | Population 1 | Emerald, Queensland | CGE00046415 |
| *H. verdcourtii* Craven | Population 2 | St. George, Queensland | CGE00046414 |
| *H. verdcourtii* Craven | Population 3 | St. George, Queensland | CGE00080882 |
| *H. verdcourtii* Craven | Population 4 | Theodore, Queensland | CGE00046413 |
| *H. verdcourtii* Craven | Population 5 | Narrabri, NSW | CGE00046366 |
| *H. tridactylites* Lindl. | Population 6 | Narrabri, NSW | CGE00046364 |
| *H. tridactylites* Lindl. | Population 7 | Jimbour, Queensland | CGE00046363 |

**Table S1.** *Hibiscus* species used in this study.

| **Line** | **Plant Background** | **Construct** | **Description** |
| --- | --- | --- | --- |
| AF29.1 | *H. trionum* | pAF29 | *HtCREAM1* overexpression |
| AF52.1 | *H. trionum* | pAF52 | *HtBERRY1* overexpression |
| VT9.17 | *H. trionum* | pVT9 | *HtDFR1* overexpression |
| VT9.6 | *H. trionum* | pVT9 | *HtDFR1* knockdown |

**Table S2.** *H. trionum* transgenic lines generated in this study.

| **Gene** | **Sequence** | **Purpose** |
| --- | --- | --- |
| HtDFR1-qF | TGCCGCCTAGCTTGATTACC | qPCR |
| HtDFR1-qR | CACGAACTGCCCTTGCCTAATA | qPCR |
| HtDFR2-qF | ACCTCCTTTAGTGGTTGGTCC | qPCR |
| HtDFR2-qR | TCCCAGTGATGGGAGAAAGTG | qPCR |
| HtDFR3-qF | TGGGACTTCTGCAACGAGAATAA | qPCR |
| HtDFR3-qR | ATCGGAGGCAGTGGAACATAAA | qPCR |
| HtFLS1-qF | GGAAGAGAAAGAGGTGTACGCTAAG | qPCR |
| HtFLS1-qR | CAGCCCAGTTCTTCTTCCCATTA | qPCR |
| HtFLS2-qF | GAATACGCTAAGCACATGCATGG | qPCR |
| HtFLS2-qR | CATGTCGTCCCCACCTAGAG | qPCR |
| HtFLS3-qF | GCGCCCTCGTTGTTCATATTG | qPCR |
| HtFLS3-qR | TCCGAGTTCTCTCCTTGTCTAC | qPCR |
| HtBERRY1-qF | GGTAGACTGCCTGGAAGAACAG | qPCR |
| HtBERRY1-qR | GATGAGGTTTCGGGTTCGAGTTA | qPCR |
| HtBERRY2-qF | GGTAGACTGCCAGGAAGAACAT | qPCR |
| HtBERRY2-qR | TGGATGAGGTTTCGGGTTTGA | qPCR |
| HtCREAM1-qF | GGACCGGGTTTCTTTCTGCTTAT | qPCR |
| HtCREAM1-qR | GTCATCTCCACTTGCTGGTACAC | qPCR |
| HrBERRY1-qF | GGTAGACTGCCTGGAAGAACAG | qPCR |
| HrBERRY1-qR | GATGAGGTTTCGGGTTCGAGTTA | qPCR |
| HrBERRY2-qF | GGTAGACTGCCAGGAAGAACAT | qPCR |
| HrBERRY2-qR | TGGATGAGGTTTCGGGTTTGA | qPCR |
| HrCREAM1-qF | GGACCGGGTTTCTTTCTGCTTAT | qPCR |
| HrCREAM1-qR | GTCATCTCCACTTGCTGGTACAC | qPCR |
| HtBERRY1-F | TTTGAGGGTTAGTGTTAAACAGCTACG | genotyping |
| HtBERRY1-R | AAACTTCTGGCTATAAGTTGAACACATTC | genotyping |
| HrBERRY1-F | CCTCGGTAACAGGTAACTCATTAG | genotyping |
| HrBERRY1-R | GGTCGAGTATGGTCCAATGG | genotyping |
| HvBERRY1-F1 | ATGGAAAGGCCATCTTTAAGTGTGA | genotyping |
| HvBERRY1-F2 | GATCAATCCTTCCGACAGTAACTCAAACC | genotyping |
| HvBERRY1-R1 | CTATAGGTTGAACACATTCCAGAACTCTGC | genotyping |
| HvBERRY1-R2 | TTGGTTGAACCTGGTTGTTGATCA | genotyping |
| HvBERRY1-R3 | GGTTTGAGTTACTGTCGGAAGGATTGATC | genotyping |
| HvACTIN1-F | CCCAGATCATGTTTGAGACCTT | genotyping |
| HvACTIN1-R | ACCGGAATCCAGCACAATAC | genotyping |

**Table S3.** Primer sequences used in this study.

| **Vector** | **Recombinant DNA** | **Reference** |
| --- | --- | --- |
| pSG55 | Modified pCAMBIA1300 with 2xp35S and pUBQ10::eYFPmyr | This study |
| pAF29 | 2xp35SS::HtCREAM1 in pSG55 | This study |
| pAF52 | 2xp35SS::HtBERRY1 in pSG55 | This study |
| pVT9 | 2xp35SS::HtDFR1 in pSG55 | This study |

**Table S4.** Plant expression vectors generated for this study.
