# Supplementary material for: The genetic basis of replicated bullseye pattern reduction across the Hibiscus trionum complex
Source: New Phytol. 2025 May 20;247(2):863–83. doi: 10.1111/nph.70168 (PMC12177274; doi:10.1111/nph.70168)
Supplement: Supplementary file 4 — Dataset S4 Complete MYB family tree generated using the 483 sequences from Dataset S3. [file NPH-247-863-s005.pdf]

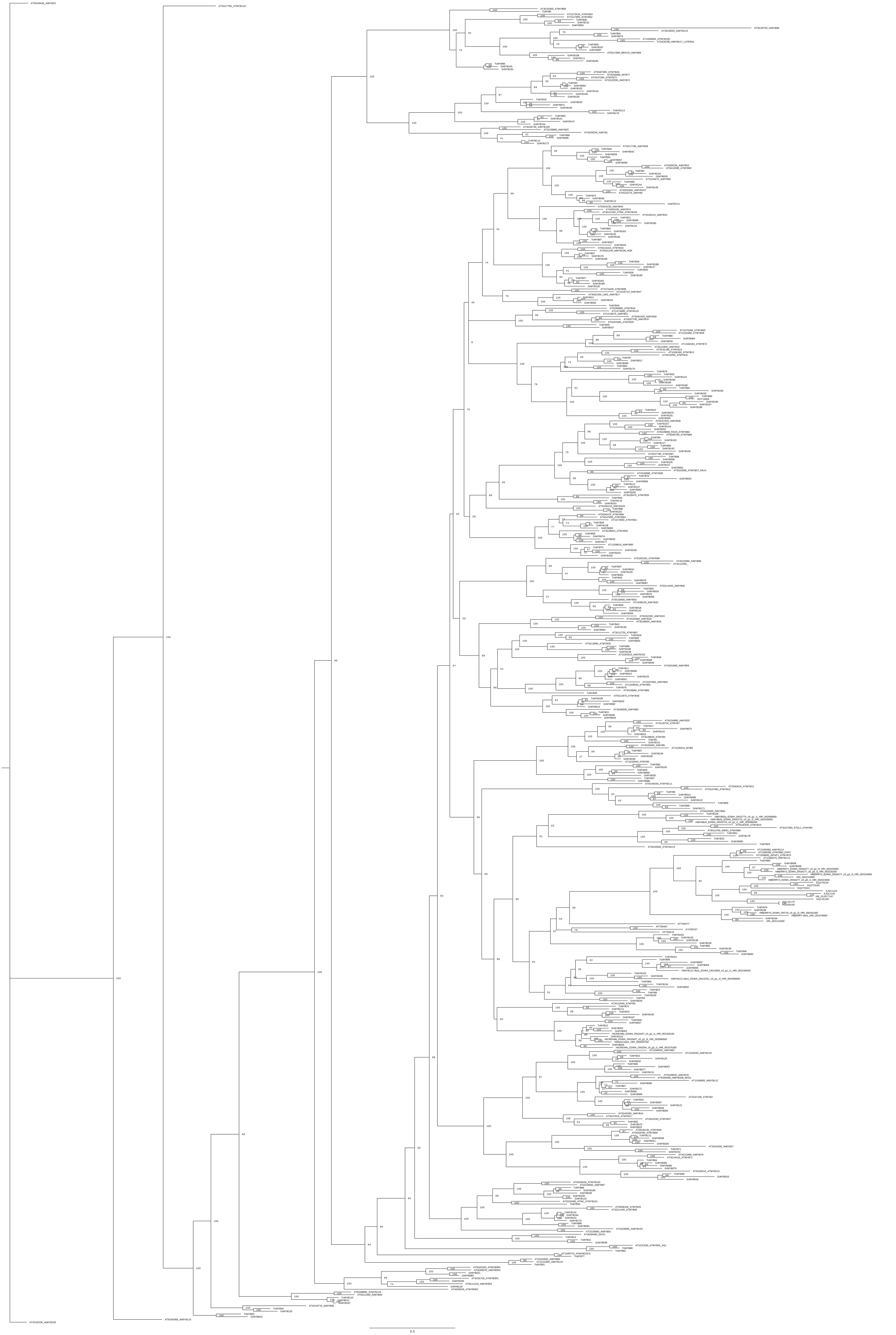
0.5
